# A Novel RNA Transcript with Antiapoptotic Function Is Silenced in Fragile X Syndrome

**DOI:** 10.1371/journal.pone.0001486

**Published:** 2008-01-23

**Authors:** Ahmad M. Khalil, Mohammad Ali Faghihi, Farzaneh Modarresi, Shaun P. Brothers, Claes Wahlestedt

**Affiliations:** Molecular and Integrative Neurosciences Department (MIND), The Scripps Research Institute, Jupiter, Florida; University of the Western Cape, South Africa

## Abstract

Several genome-wide transcriptomics efforts have shown that a large percentage of the mammalian genome is transcribed into RNAs, however, only a small percentage (1–2%) of these RNAs is translated into proteins. Currently there is an intense interest in characterizing the function of the different classes of noncoding RNAs and their relevance to human disease. Using genomic approaches we discovered *FMR4*, a primate-specific noncoding RNA transcript (2.4 kb) that resides upstream and likely shares a bidirectional promoter with *FMR1*. *FMR4* is a product of RNA polymerase II and has a similar half-life to *FMR1.* The CGG expansion in the 5′ UTR of *FMR1* appears to affect transcription in both directions as we found *FMR4*, similar to *FMR1*, to be silenced in fragile X patients and up-regulated in premutation carriers. Knockdown of *FMR4* by several siRNAs did not affect *FMR1* expression, nor *vice versa*, suggesting that *FMR4* is not a direct regulatory transcript for *FMR1*. However, *FMR4* markedly affected human cell proliferation *in vitro*; siRNAs knockdown of *FMR4* resulted in alterations in the cell cycle and increased apoptosis, while the overexpression of *FMR4* caused an increase in cell proliferation. Collectively, our results demonstrate an antiapoptotic function of *FMR4* and provide evidence that a well-studied genomic locus can show unexpected functional complexity. It cannot be excluded that altered *FMR4* expression might contribute to aspects of the clinical presentation of fragile X syndrome and/or related disorders.

## Introduction

While at least 40–50% of the human genome is transcribed into RNA, only 1.2% of the genome is translated into protein [1]–[3]. RNAs which do not code for proteins (noncoding RNAs) have been classified into different classes (tRNA, rRNA, snRNA, snoRNA, miRNA, siRNA, piRNA, natural antisense transcripts and long noncoding RNA) based on their size and function. Novel classes of noncoding RNAs have been shown to have a variety of functions which include translational inhibition (miRNA) [4], mRNA degradation (siRNA) [5], and repressing transposition (piRNA) [6], [7]. Also, thousands of protein coding genes have now been shown to have antisense transcripts [8]. Antisense transcript manipulation can in some cases lead to the repression of the sense transcript (discordant regulation) or enhance the stability of the sense transcript (concordant regulation) [8]. However, the exact mechanisms by which antisense transcripts regulate their sense partners are likely diverse and require further studies. Another class of noncoding RNAs is long noncoding RNAs (or “macroRNA”) [9], [10] which do not overlap with protein-coding genes and range from 300 nucleotides to over 10 kb in size with an average size of ∼2 kb [9], [11]. Notably, the sequence of noncoding RNAs, in contrast to other noncoding RNAs such as miRNAs and snoRNAs, is often not well conserved even between mammals [9] and can function both *in cis* (*e.g*., *XIST*) [12] and *in trans* (e.g., *HOTAIR*) [13]. In contrast to other noncoding RNAs, only a small number of long noncoding RNAs have been functionally characterized. Several studies have now shown that the expression pattern of noncoding RNAs can be altered in several human diseases such as cancers and heart disease [14], [15] suggesting that noncoding RNAs may have a functional relevance to human disease and that they may be considered as potential drug targets [11], [16].

Fragile X syndrome (FXS), the most common cause of inherited mental retardation, is caused by the expansion of CGG trinucleotide repeats in the 5′ UTR of the fragile X mental retardation 1 gene (*FMR1*) [17]–[19]. Normal individuals have a range of 5–50 CGG repeats in the 5′ UTR of *FMR1* and express *FMR1* in a wide range of adult and embryonic tissues [20]. The CGG repeats can expand in the female germ line or shortly after fertilization by an unknown mechanism. Individuals with 55–200 repeats are premutation carriers and generally express higher levels of *FMR1* mRNA than normal individuals and may result in a clinical condition termed fragile X tremor and ataxia syndrome (FXTAS) [21], [22]. The expansion of CGG repeats above 200 leads to the repression or silencing of *FMR1* and consequently to the absence of the fragile X mental retardation protein (FMRP). We have previously been part of effort to identify and characterize 2,113 bidirectional promoters from 42,887 transcriptional units in humans [23]. In the present manuscript, we report the discovery and functional characterization of a primate-specific noncoding RNA (*FMR4*) that becomes silenced as a result of the CGG expansion in the 5′ UTR of *FMR1* in fragile X syndrome. *In vitro* manipulation of *FMR4* indicates it has an antiapoptotic function in human cells.

## Results

### Identification and expression analysis of *FMR4*

**Figure 1:**
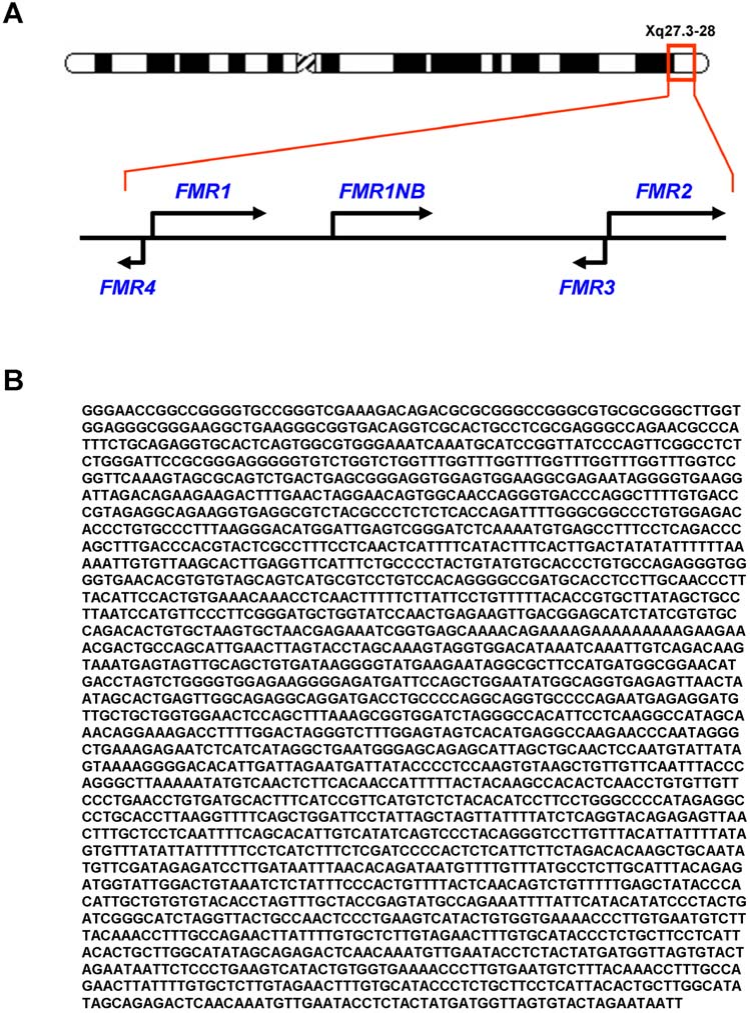
Identification and sequence analysis of *FMR4*. (A) Schematic showing known genes in Xq27.3-28 including the newly identified *FMR4*. *FMR4* is transcribed upstream of *FMR1* and in the opposite direction. (B) Sequence of *FMR4* obtained by *r*apid *a*mplification of *c*DNA *e*nds (RACE).

Previous work by us and others has shown that bidirectional promoters are relatively common in the mammalian genome [8], [23]. Therefore, using genomic approaches, including rapid amplification of cDNA ends (RACE), regular and real time PCR (RT-PCR), we searched for transcripts upstream of *FMR1* that could also be affected by the CGG repeat expansion. Here, we report the identification of a novel 2.4 kb long noncoding RNA, which we named *FMR4*, that resides upstream and likely shares a bidirectional promoter with *FMR1* (Figure 1A–B). Bioinformatic analysis suggests that *FMR4* does not have a conventional open reading frame, to confirm that *FMR4* is indeed a noncoding RNA we carried out *in vitro* transcription/translation followed by mass spectrometry analysis; however, no protein was detected suggesting that *FMR4* is most likely a noncoding RNA (data not shown). Northern blot analysis shows that *FMR1* is expressed in the majority of the human tissues examined consistent with previous reports [20]. *FMR4* is expressed in several adult human tissues including brain, liver, placenta, small intestine, colon and spleen but not in the pancreas, testes, ovaries or prostate (Figure S1). Two bands corresponding to *FMR4* were observed in several human adult tissues, one possibility is that there is alternative transcription start sites for *FMR4*, however, when we performed our RACE analysis we used RACE ready cDNA from SH-SY5Y cells and obtained only the longer 2.4 kb transcript.

### *FMR4* is ubiquitously expressed during human development

**Figure 2:**
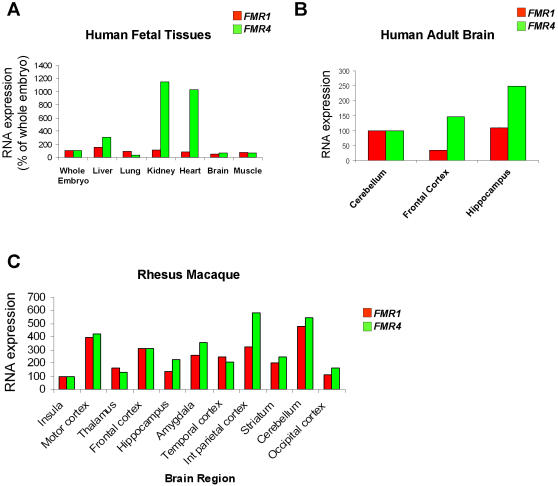
Expression analysis of *FMR4*. (A) RT-PCR analysis of *FMR4* and *FMR1* in seven different human fetal tissues (week 12), RNA from each tissue was pooled from at least three fetuses (GBiosciences). The RNA expression of *FMR1* and *FMR4* were normalized to whole embryo (set as 100%). Both transcripts are expressed in all the tissues tested with notably high expression of *FMR4* in the kidney and heart. (B) RNA was extracted from six postmortem human adult brains from three different regions, thereafter; cDNA synthesis followed by RT-PCR was performed on all samples to measure the relative quantities of *FMR1* and *FMR4*. Both *FMR1* and *FMR4* are highly expressed in all the human brain regions tested. (C) RT-PCR analysis of *FMR4* and *FMR1* in several regions of two monkeys brains. The RNA expression of *FMR4* and *FMR1* were normalized to the insula (set as 100%).

Since *FMR4* is expressed in several human adult tissues, we next examined its expression levels in human fetal tissues. Using RT-PCR we measured RNA expression levels of *FMR4* in seven different human fetal tissues (12 weeks); the RNA from each tissue was pooled from at least three different embryos (GBiosciences). We found *FMR4* to be expressed in all the tissues examined including the brain. Notably, *FMR4* is highly expressed in the kidney and heart at that stage of human development (Figure 2A). The high expression of *FMR4* in the heart is consistent with a previous report that patients with fragile X syndrome have cardiac defects similar to those seen in other disorders of connective tissue such as Marfan's syndrome and Ehlers-Danlos syndrome [24].

### *FMR4* is expressed in human and monkey brain

To determine whether *FMR4* shows differential expression within different regions of the human brain, we examined the RNA concentrations of both *FMR1* and *FMR4* by Real-Time PCR (RT-PCR) using tissue from six postmortem human brains (from four males and two females aged 61–91 years) and studied three different regions (cerebellum, frontal cortex, and hippocampus). By this quantitative method, RT-PCR, both *FMR1* and *FMR4* were shown to display robust expression levels in all three brain regions tested (Figure 2B). To determine if *FMR4* is also expressed in other primates, we examined the expression of *FMR4* in rhesus monkey brain regions using RT-PCR. Total RNA from each brain region was isolated from two monkeys and DNAse treated prior to cDNA synthesis. We found *FMR4* to be expressed in all the monkey brain regions tested with high expression in the cerebellum and interior parietal cortex (Figure 2C) confirming that *FMR4* is expressed in other primates in addition to humans.

### The CGG expansion affects transcription in both directions of a bidirectional promoter

**Figure 3:**
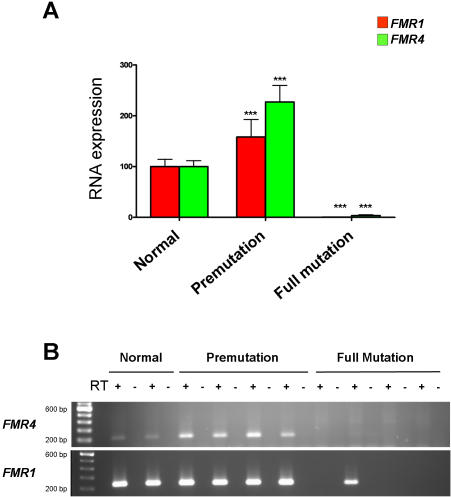
*FMR4* is silenced in fragile X syndrome. (A) RNA from four normal, four premutation and four full mutation FXS patients isolated from untransformed leucocytes (kindly provided by Flora Tassone and Paul Hagerman, UC Davis) was reverse transcribed using random hexamers. Quantitative RT-PCR analysis revealed that *FMR4*, similar to *FMR1*, is up-regulated in pre-mutation carriers and shut down in full mutation fragile X patients (P<0.0001). (B) RNA from untransformed leucocytes were reversed transcribed and the cDNA was used for PCR analysis. *FMR4* is expressed in normal and premutation carriers but no bands were observed in the full mutation fragile X patients (35 cycles). *FMR1* bands were observed in normal, premutation, and one of the full mutation patients (35 cycles). To account for any possible DNA contamination, no reverse transcriptase control for all samples were used in the PCR (lanes next to bands are all negative indicating no DNA contamination was present). Error bars: s.d.

To determine if the expression of *FMR4* is affected by the CGG expansion in the 5′ UTR of *FMR1* that occurs in FXS and/or FXTAS, we investigated the relative expression of *FMR4* and *FMR1* by RT-PCR in untransformed leukocytes from four control, four premutation and four FXS patients. We found *FMR4* expression, similar to *FMR1*, to be significantly up-regulated in premutation carriers, and shut down in full mutation (FXS) patients (P<0.0001) (Figure 3A). All the samples tested had a matching control without the reverse transcriptase to account for possible DNA contamination. We also utilized regular PCR (35 cycles), gel electrophoresis and ethidium bromide staining to examine the expression of *FMR4* and *FMR1* from the same samples. *FMR4* was detectable in both the normal and premutation carriers but not in the full mutation fragile X patients. *FMR1* was detectable in the normal, premutation carriers and one out of the four fragile X patients (Figure 3B); this is not surprising since variable levels of *FMR1* mRNA have been previously shown to be present in some fragile X patients [25].

### *FMR4* is a product of RNA polymerase II and has a similar half-life to *FMR1*

**Figure 4:**
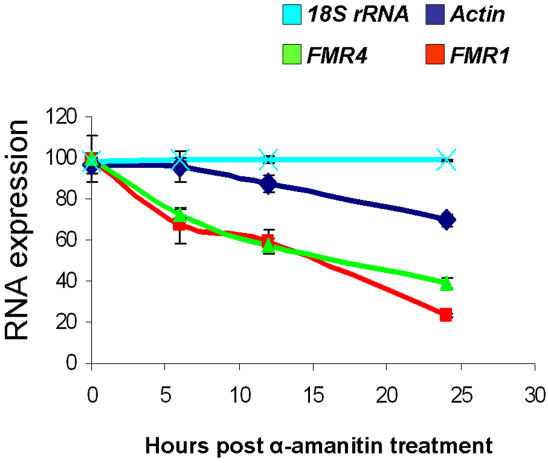
*FMR4* has a similar half-life to *FMR1*. HEK-293T cells were treated with α-amanitin (blocks RNA polymerase II) and the levels of *FMR4* and *FMR1* were measured by RT-PCR at 0, 6, 12 and 24 hours post treatment. Both *FMR4* and *FMR1* have similar half-lives. These experiments also further confirm that *FMR4* is a product of RNA polymerase II.

To measure the relative half-lives of *FMR1* and *FMR4* we treated HEK-293T cells with 50 µM of α-amanitin (an inhibitor of RNA polymerase II) [26] and RNA was isolated at 0, 6, 12 and 24 hours post treatment (six repeats each). All treated samples had a matching control which did not receive α-amanitin (untreated samples). *Actin* was used as a positive control and *18S* rRNA was used as a negative control (a product of RNA polymerase I). By RT-PCR we measured the levels of *Actin*, *18S*, *FMR1*, and *FMR4*. As expected *18S* rRNA levels did not change at any of the time points tested since α-amanitin does not affect RNA polymerase I. By contrast *Actin*, *FMR1* and *FMR4* were all affected since they are all products of RNA polymerase II. The data indicates that *FMR4* has a similar half-life to *FMR1* (Figure 4).

### No evidence of direct cross-regulation between *FMR1* and *FMR4*

**Figure 5:**
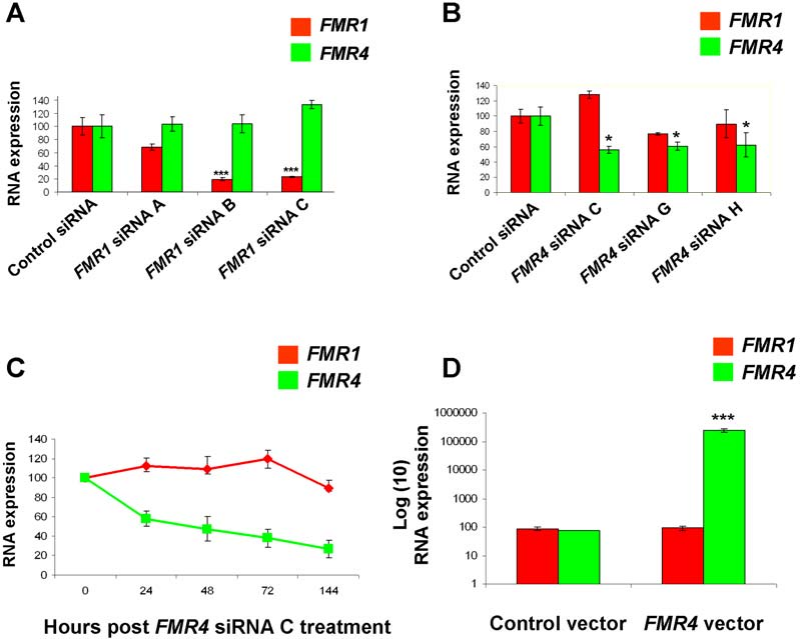
No direct cross-regulation between *FMR1* and *FMR4.* (A) We used three distinct siRNAs against *FMR1* to transfect HEK-293T cells. Two out of the three siRNAs resulted in a significant knockdown of *FMR1* (80%), but did not affect *FMR4* RNA levels. (B) We used three distinct siRNAs against *FMR4* to transfect HEK-293T cells. All three siRNAs resulted in a significant knockdown of *FMR4* but did not affect *FMR1* RNA levels. (C) Significant knockdown of *FMR4* via siRNA C did not result in a change in *FMR1* RNA levels at any of the time points tested (24, 48, 72, or 144 hours post transfection). (D) The entire sequence of *FMR4* was cloned into a pcDNA3.1 vector with a CMV promoter. The pcDNA3.1 vector containing the *FMR4* sequence and the original pcDNA3.1 (without the *FMR4* insert) were transfected in HEK-293T cells. At 72 hours post transfection, RNA was isolated and reversed transcribed and used for RT-PCR analysis. There is a highly significant increase in the *FMR4* RNA levels but no effect on *FMR1* RNA. Error bars: s.d.

To determine whether *FMR1* and *FMR4* are functionally linked, we tested three different siRNAs against *FMR1* and identified three different newly designed siRNAs against *FMR4*. It is important to utilize multiple efficacious siRNAs to any given target in order to avoid the possibility of off-target phenomena [11], [27]. Also, since *FMR4* is highly expressed in human embryonic kidney (Figure 2A), we decided to use HEK-293T cells as an *in vitro* system to study *FMR4* regulation and function. First, we tested three siRNAs against *FMR1* by transfecting HEK-293T cells with 20 nM (final concentration) of *FMR1* siRNAs (six repeats each). Two out of the three siRNAs tested were effective in knocking down *FMR1* by 80% as early as 48 hours post transfection (siRNA B and C, Table 1). This level of knockdown was also observed at 72 hours post transfection and in repeated transfection experiments (144 hours total, two transfections at 0 hour and 72 hours post first transfection). However, in all cases the concentrations of *FMR4* were not affected by *FMR1* siRNAs (Figure 5A). We designed and tested nine different siRNAs for *FMR4*, three siRNAs (C, G, and H, see methods for sequences) caused a significant decrease in *FMR4* at 48 and 72 hours post transfection, but did not affect the levels of *FMR1* (Figure 5B). We used *FMR4* siRNA C which caused the highest level of *FMR4* knockdown among the siRNAs tested for a time course experiment. HEK-293T cells were transfected with *FMR4* siRNA C and RNA was collected at 24, 48, 72 and 144 hours post transfection. This siRNA caused a significant knockdown of *FMR4* but did not affect *FMR1* levels at any of the time points tested (Figure 5C). We next cloned *FMR4* into a pcDNA3.1 vector with a CMV promoter and overexpressed *FMR4* in HEK-293T cells, a pcDNA3.1 vector without the *FMR4* insert was used as a control. At 72 hours post transfection, RNA was isolated, reversed transcribed and used for RT-PCR analysis. As expected, *FMR4* overexpression led to a substantial increase in *FMR4* RNA levels, but did not have any effect on *FMR1* RNA (Figure 5D). Collectively, these experiments suggest that the non-overlapping transcripts *FMR1* and *FMR4* do not regulate each other.

**Table 1:** Sequences of *FMR1* and *FMR4* siRNAs.

siRNA	Sequence	Supplier
***FMR1*** ** A**	GGUGUAUUCCAGAGCAAAUtt	Ambion (ID # 10824)
***FMR1*** ** B**	GGGUGAGUUUUAUGUGAUAtt	Ambion (ID # 11010)
***FMR1*** ** C**	GGAUGAUAAAGGGUGAGUUtt	Ambion (ID # 10919)
***FMR4*** ** A**	GCCCUCUCUCACCAGAUUUtt	QIAGEN
***FMR4*** ** B**	AGGGCCAGAACGCCCAUUUtt	QIAGEN
***FMR4*** ** C**	GUGGCGUGGGAAAUCAAAUtt	QIAGEN
***FMR4*** ** D**	GCAUCCGGUUAUCCCAGUUtt	Invitrogen
***FMR4*** ** E**	UCGCCUUUCCUCAACUCAUtt	Invitrogen
***FMR4*** ** F**	GCACUUGAGGUUCAUUUCUtt	Invitrogen
***FMR4*** ** G**	AGAGAUCCUUGAUAAUUUAtt	QIAGEN
***FMR4*** ** H**	CAUUGAUUAGAAUGAUUAUtt	QIAGEN
***FMR4*** ** I**	GUAGGUGGACAUAAAUCAAtt	QIAGEN

### *FMR4* affects cell proliferation in human cells

**Figure 6:**
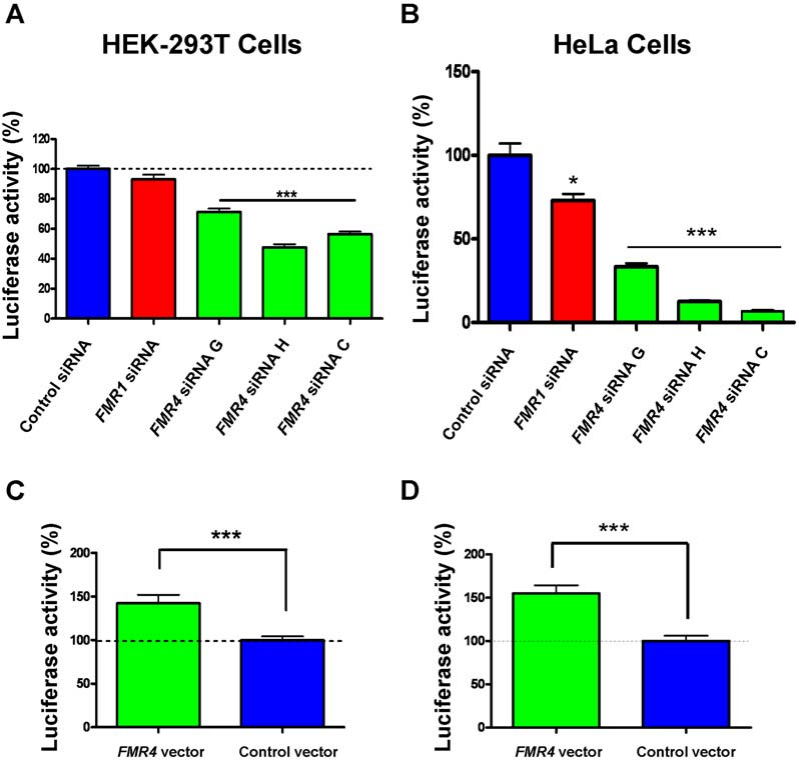
*FMR4* affects proliferation in human cells. (A) Cell proliferation assay showing that the knockdown of *FMR4* via three distinct siRNAs in HEK-293T cells, but not knockdown of *FMR1*, resulted in decrease in cell proliferation in comparison to cells which are treated with a negative control siRNA. Cell proliferation was measured based on luciferase activity in these cells at 72 hours post siRNA transfection. (B) Cell proliferation assay showing that the knockdown of *FMR4* via three distinct siRNAs in HeLa cells resulted in decrease in cell proliferation in comparison to cells which are treated with a negative control siRNA (P<0.0001). (C–D) In both HEK-293T and HeLa cells, overexpression of *FMR4* resulted in an increase in cell proliferation in comparison to cells treated with a control vector. Error bars: s.d.

**Figure 7:**
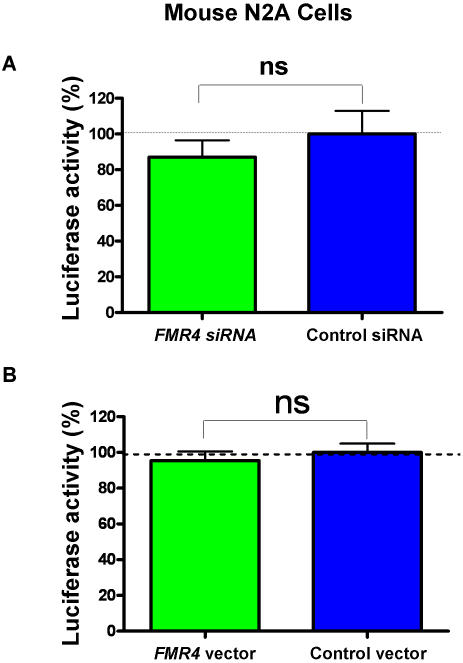
The effect of *FMR4* on cell proliferation is not observed in non-primates. Since *FMR4* is a primate-specific transcript we examined its effect on cell proliferation in non-primates using mouse N2A cells. We examined both the siRNA knockdown of *FMR4* (as a negative control experiment) and the over-expression of *FMR4* on cell proliferation in N2A cells. (A) Mouse N2A cells were transfected with *FMR4* siRNA C and a control siRNA. Simultaneously, cells were transfected with pGL3 (luciferase) vector. At 72 hours post transfection luciferase activity was measured and data are graphed as a percentage of control siRNA. (B) Mouse N2A cells were transfected with *FMR4* over-expression vector and a control vector (no *FMR4* insert). Simultaneously, cells were transfected with pGL3 (luciferase) vector. At 72 hours post transfection luciferase activity was measured and data are graphed as a percentage of control vector. Unlike human cells which show an increase in cell proliferation when transfected with the *FMR4* vector, mouse N2A cells did not show any change in proliferation.

To uncover a possible function for *FMR4*, we examined the effects of three distinct and efficacious siRNAs against *FMR4* on cell proliferation using a luciferase reporter system (see methods). HEK-293T cells were transfected with either siRNAs targeting *FMR4* (C, G, and H), *FMR1, or* a control siRNA. All cells were simultaneously co-transfected with a pGL3 (luciferase) vector. At 72 hours post transfection, luciferase activity, which is a marker of cell proliferation, was measured using an Analyst GT Multimode Reader (Molecular Devices) and all data points were plotted as a percentage of control siRNA treated cells. All three siRNAs against *FMR4*, but not the siRNA against *FMR1*, resulted in significant decreases in cell proliferation in comparison to the control siRNA treated cells (Figure 6A). To determine if the effect of *FMR4* on cell proliferation is reproducible in other human cell lines we carried out a similar experiment using HeLa cells and found that all three siRNAs against *FMR4* also resulted in highly significant decreases in cell proliferation in comparison to control siRNA (P<0.0001) (Figure 6B). In contrast to HEK-293T cells, *FMR1* siRNA resulted in a marginally significant decrease in cell proliferation in HeLa cells (Figure 6B). We also carried out a similar experiment using mouse N2A neuroblastoma cells as a negative control experiment and as expected siRNAs against *FMR4* had no effect on cell proliferation in mouse N2A cells (Figure 7A). To determine if the overexpression of *FMR4* have an opposite effect compared to *FMR4* siRNAs knockdown on cell proliferation, we next transfected HEK-293T and HeLa cells with *FMR4* overexpression vector. We found that the overexpression of *FMR4* in both cell lines resulted in an increase in cell proliferation compared to the control vector treated cells (P<0.0001) (Figure 6C–D). The overexpression of *FMR4* in mouse N2A cells had no effect on cell proliferation (Figure 7B).

### *FMR4* has antiapoptotic properties

**Figure 8:**
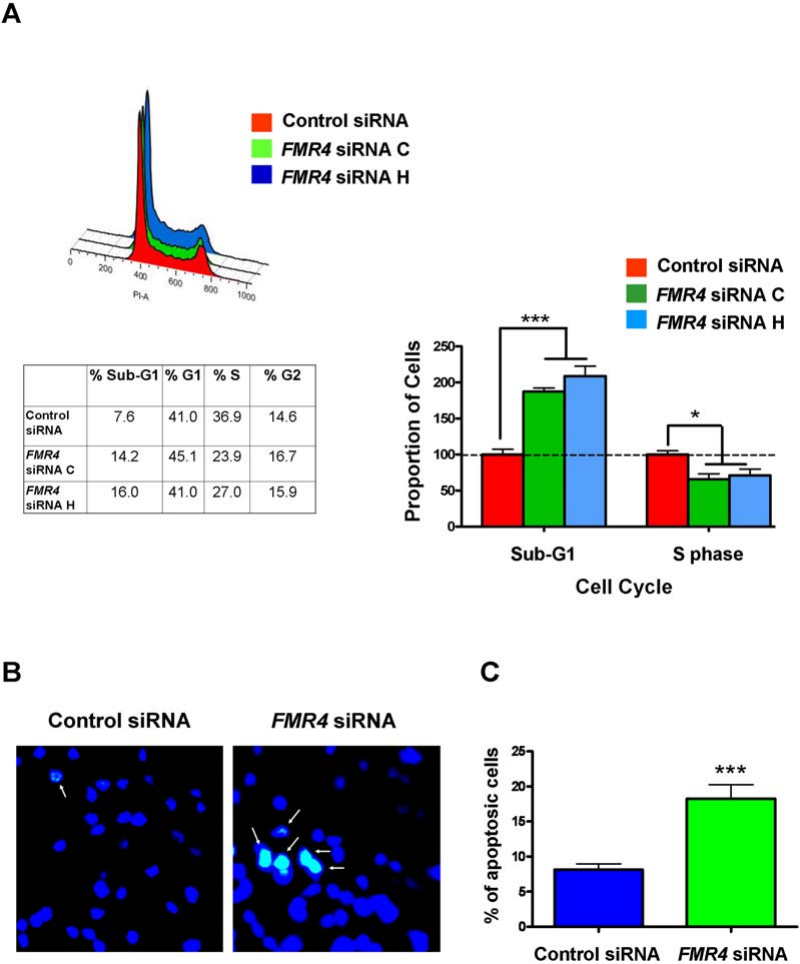
*FMR4* has an antiapoptotic function in human cells. (A) Cell cycle analysis of control cells (red) and cells treated with two different siRNAs against *FMR4* (green and blue) shows that knockdown of *FMR4* resulted in a highly significant increase in the number of cells in Sub-G1 and a modest but significant decrease in the number of cells in the S phase suggesting a possible role in apoptosis. (B) Microscope images of cells (DAPI stained) treated with a control siRNA and cells treated with *FMR4* siRNA for 72 hours prior to a TUNEL assay. A significant number of cells are undergoing apoptosis (FITC) in the *FMR4* siRNA treated cells in comparison to the control siRNA treated cells. (C) Quantification of cells following a TUNEL assay indicated that there is at least a two-fold change in the number of cells undergoing apoptosis in the *FMR4* siRNA treated cells in comparison to the control siRNA treated cells. Error bars: s.d.

To further characterize the manner in which *FMR4* affects cell proliferation, we knocked down *FMR4* using two different siRNAs (four repeats each), and we used a control negative siRNA (four repeats also) to examine the effects of *FMR4* on the cell cycle in HEK-293T cells. At 72 hours post transfection, we performed propidium iodide FACS analysis (see methods) and found that the siRNAs knockdown of *FMR4* resulted in an increase in the number of cells in the Sub-G1 phase and a modest but significant decrease in the number of cells in S phase of the cell cycle (Figure 8A). This suggested that *FMR4* may have an antiapoptotic function in human cells; therefore, we next performed a fluorescent TUNEL assay and found a significant increase in apoptosis in cells treated with *FMR4* siRNA (72 hours post transfection) compared to cells treated with a control siRNA, further indicating that *FMR4* has an antiapoptotic function in human cells (Figure 8B–C).

## Discussion

In the present manuscript we report the discovery of a 2.4 kb noncoding RNA (*FMR4*) which is transcribed upstream of *FMR1*. There is no overlap between the *FMR1* and *FMR4* transcripts, and therefore, *FMR4* is not a natural antisense transcript to *FMR1*. *FMR4* is expressed in human adult and fetal tissues, and in several regions of human and rhesus monkey adult brain but at varying concentrations. Despite the likelihood that *FMR4* shares a bidirectional promoter with *FMR1, FMR4* is not expressed in the adult testes, ovary and prostate where *FMR1* is highly expressed. It is possible however that *FMR4* is expressed in these tissues during embryonic and/or fetal development as the RNAs used in our experiments from these tissues (testes, ovary, and prostate) were obtained from human adults. Notably, we found *FMR4* to be highly expressed in fetal heart and kidney. The cardiac expression of *FMR4* may possibly be of functional relevance considering the fact that many patients with fragile X syndrome exhibit heart defects such as dilation of the aortic root and mitral valve prolapse [24], [28]. Moreover the high expression of *FMR4* in the kidney (Figure 2A) appears consistent with our observations that the human embryonic kidney cell line, HEK-293, also expresses *FMR4.*

Bioinformatics analysis shows that the genomic sequence encompassing *FMR4* is conserved in other primates (chimp and rhesus monkey) with only partial homology to the mouse (Figure S2). Interestingly, however, there is an apparent transcript in the mouse X chromosome that is on the minus strand that starts approximately 100 bp upstream of the mouse *Fmr1* gene. This transcript (NCBI accession number AK148387) does not have significant homology with the human *FMR4* transcript (∼3% nucleotide identity by ClustalW using default analysis parameters for nucleotide sequences). Furthermore, this mouse transcript appears to be highly spliced and contains 4 exons, which is an additional distinction from the human *FMR4* transcript. However, we can not rule out that *FMR4* and AK148387, despite their genomic differences, still perform a similar function. The majority of noncoding RNAs identified to date seem to be poorly conserved even among mammals; this is in contrast to other noncoding RNAs (*e.g*., microRNAs and snoRNAs) which show a high level of conservation among diverse species [9]. It is worth noting that while the human and mouse *Xist* show 66% homology [29]; however, there seems to be rapid evolution of unique sequences [30]. Also, several attempts to find an orthologue for *XIST* in marsupials have failed [31], [32] despite the fact that imprinted X inactivation still occurs in marsupials, a process that requires *XIST* in eutharians [33] suggesting the possibility that a non-conserved noncoding RNA in marsupials may perform a similar function to *XIST*.

We found that *FMR4* regulates human cell proliferation *in vitro*; knockdown of *FMR4* resulted in alterations in the cell cycle and apoptosis while the overexpression of *FMR4* leads to an increase in cell proliferation. While the involvement of poorly studied protein coding gene(s) within the *FMR1* genomic locus cannot be excluded at this time, our results suggest that the *FMR4* function involves a noncoding mechanism. Recently, a long noncoding RNA, similar in size to *FMR4*, was identified in the *HOXC* locus (*HOTAIR*) [13]. The *HOTAIR* noncoding RNA represses transcription *in trans* across 40 kb of the *HOXD* locus by altering the chromatin modifications through enhancement of the PCR2 activity at the *HOXD* locus [13]. It is therefore possible that *FMR4* may also target a set of genes *in trans* resulting in its antiapoptotic properties. In conclusion, our findings should add further evidence that novel nonconserved noncoding RNAs may be functional and that they do not simply represent “transcriptional noise”.

While our manuscript was under review, Ladd *et al.* published on an antisense transcript that spans the CGG repeats in the 5′ UTR of *FMR1* [34]. This transcript, *ASFMR1*, is up-regulated in premutation carriers and shut down in fragile X patients similar to *FMR1* and *FMR4*. Also, one splice variant of *ASFMR1* overlaps with *FMR4*, therefore it is possible that *FMR4* is nested in the 3′ UTR of *ASFMR1*, a phenomenon seems to be prevalent in many genes throughout mammalian genomes [1] or another possibility is that these two transcripts may also have contiguous isoforms which is another intriguing possibility. *ASFMR1* appears to be highly spliced and has multiple transcription start sites. One transcription start site is in intron 2 of *FMR1* at position +10243. This transcription start site was identified by utilizing a 5′ RACE primer in exon 1 of *FMR1*. Also, multiple transcription start sites for *ASFMR1* were also identified in −99 to −208 upstream of *FMR1* by designing 5′ RACE primers in the −1000 position relative to *FMR1*. From these experiments it is not clear if the transcript which starts in the +10243 is the same transcript as the one that initiates in the −99 to −208. If these two transcripts were both *ASFMR1*, the authors should have been able to clone the longer transcript which initiates at +10243 using the 5′ RACE primers which they used to identify the multiple transcription start sites in the −99 to −208 positions. Therefore, another possibility is that one of the transcripts that Ladd *et al.* cloned and identified as an alternative transcript to *ASFMR1* is the *FMR4* transcript. In essence, our findings along with Ladd *et al.* suggest a complex transcription within the *FMR1* locus than previously thought and should provide an incentive for further studies to determine the exact nature of all the transcripts, and their function, throughout the *FMR1* region and their relevance to *FMR1*-associated human disorders (fragile X syndrome and FXTAS).

## Materials and Methods

### Rapid amplification of cDNA ends (RACE)

The genomic sequence for *FMR1* locus was obtained from the UCSC website (http://genome.ucsc.edu/). RACE-ready cDNA (0.5 ng/µl) from SH-SY5Y cells for 5′ and 3′ RACE was custom made by Ambion (Austin, TX). RACE primers were designed using Primer3 software and the name and sequence of the RACE primers for *FMR4* is listed here: *FMR4* 5′ outer 1: TGAGTTGAGGAAAGGCGAGT; *FMR4* 5′ inner 1: TTGAGATCCCGACTCAATCC. We carried out two rounds of PCR using the outer and inner primers sequentially. First PCR was carried out using 10 µl of RACE-ready cDNA and the second PCR was carried out using 2 µl of the first PCR product. PCR conditions were as follow: 94 C for 5 minutes, (94 C for 30 seconds, 59 C for 30 seconds, 72 C for 2 minutes) for 35 cycles, 72 C for 10 minutes. Both first and second PCR products were ran on a 2% agarose gel and bands of interest were cut, purified, cloned into pGEM T-easy vector (Promega, Madison, WI) before sequencing (UC Davis Sequencing Core Facility).

### RNA Extraction and cDNA synthesis

Total RNA was extracted using Quiagen RNeasy mini kit (catalogue # 74106). RNA concentrations were measured using The NanoDrop® ND-1000 UV-Vis Spectrophotometer. Equal amounts of RNA were reversed transcribed using TaqMan reverse transcription reagents (part # N808-0234) according to the manufacturer's protocol.

### Real-Time PCR

*FMR4* forward primer: ACACCCTGTGCCCTTTAAGG, *FMR4* reverse primer: TCAAAGCTGGGTCTGAGGAAAG, Reporter (probe): TCGGGATCTCAAAATGT. Real-Time PCR (RT-PCR) was carried out with the GeneAmp 7900 (Applied Biosystems, Foster City, CA). The PCR reactions contained 20–40 ng cDNA, Universal Mastermix (Applied Biosystems, Foster City, CA), 300 nM of forward and reverse primers, and 200 nM of probe in a final reaction volume of 15 µl. The primers and probe were designed using File-Builder software (Applied Biosystems, Foster City, CA). The PCR conditions were as follows: 50 C for 2 min then 95 C for 10 min then 40 cycles of 95 C for 15 s and 60 C for 1 min. The results are based on cycle threshold (Ct) values. Differences between the Ct values for experimental and reference genes (18srRNA) were calculated as ΔΔCt.

### *In vitro* transcription and translation

1 ug of pcDNA3.1 containing the *FMR4* sequence (or without the sequence as a control) was utilized for *in vitro* transcription using a MAXIscript kit (Cat # AM1200, Ambion, Austin, TX) according to manufacturer's instructions. Subsequently, *in vitro* translation was carried out using the Retic Lysate IVT kit (Ambion, Austin, TX) according to manufacturer's instructions, except that Fluorotect Green-Lys (Promega, Madison, WI) was included in the translation mixture according to manufacturer's suggestions. Fluorotect Green-Lys, is a BIODPY labeled lysine that allows the addition of a fluorescent amino acid to any newly synthesized peptide (unlabeled lysine is also included in the reaction mixture) allowing for fluorescent detection of such peptides.

#### Northern blot analysis

We purchased a human ready-to-hybridize northern blot membrane from Ambion (cat# 3141) which has 2 µg of poly(A) RNA per lane isolated from human brain, liver, placenta, small intestine, colon, pancreas, spleen, prostate, testes, and ovary. The membrane was initially incubated with 15 ml of pre-warmed hybridization solution (Ambion, cat#8670) for 1 hour at 65 C. Northern blot probes (^32^P) were generated using Amersham rediprime II random prime labeling system (RPN1633) according to the manufacturer's protocol (GE Healthcare, Piscataway, NJ). We added 14 µl of the probe per 5 ml of hybridization buffer (42 µl total) to the membrane for overnight incubation at 42 C. Two low-stringency and two high-stringency washes were performed for 15 minutes each prior to exposing the membrane for phosphor imager.

#### Cell culture, siRNA transfection and RNA isolation

HEK-293T and N2A cells were cultured in MEM plus 10% FBS. Cells in the logarithmic growth phase were transfected with 20 nM of siRNA using 0.2% Lipofectamine 2000 according to manufacturer's instructions (Invitrogen, Carlsbad, CA). Cells were incubated for 72 h or transfected a second time for an additional 72 hours prior to RNA isolation. RNA was isolated using QIAGEN RNeasy mini-kit (QIAGEN, Valencia, CA). All samples were treated with RNAse-free DNase (QIAGEN, Valencia, CA, #79254) for 20 minutes as described in the manufacturer's protocol. The sequence of *FMR1* and *FMR4* siRNAs are listed in table 1.

#### *FMR4* over-expression

The entire cDNA sequence of *FMR4* was cloned into pcDNA3.1 vector. The vector was sequenced to verify the insertion of the *FMR4* sequence. The vector was transfected into several human and mouse cell lines using standard procedures.

#### Stability and α-amanitin treatment

HEK-293T cells were cultured in 6 well plates. Twenty-four h later, cells were treated with 50 µg/ml of α-amanitin (Sigma, St. Louis, MO). Cells were harvested for RNA isolation and RT-PCR at 0, 6, 12, and 24 hours post treatment. Three independent samples were taken for each data point and all samples had untreated matching samples for RNA purification and data analysis.

#### Cell proliferation assay

Using Multidrop 384Titan, 50 ng of PGL3 vector (luciferase vector with SV40 promoter), 20 nM of siRNA and transfection reagents (Lipofectamine 2000 0.2% and OptiMEM, Invitrogen, CA) were plated in 96 well plates. Equal number of cells (20,000 per well) were added to each well and incubated at 37°C for 72 hours. Bright-Glo luciferase reagent (Promega, Madison, WI) was added to each well and incubated at room temperature for 5 minutes. Luciferase activity, as a marker of cell proliferation, was measured by Analyst GT Multimode Reader (Molecular Devices, Sunnyvale, CA) and plotted against control siRNA.

#### Cell cycle analysis

We knocked down *FMR4* using two different siRNAs (4 repeats each), and we used a control negative siRNA (4 repeats also) to examine the effects of *FMR4* on the cell cycle. At 72 hours post transfection, we prepared the cells for flow cytometry as follow: cells were washed with PBS, trypsynized and centrifuged at 1,000 rpm for 10 minutes. Then the cells were washed again with PBS before being fixed with 70% ethanol at −20C overnight. The next day the cells were centrifuged, washed with PBS and re-suspended in 38 mM sodium citrate, 69 µM propidium iodide and 19 µg/ml RNAse A for flow cytometry analysis. Results were analyzed using FlowJo analysis software.

#### TUNEL assay

HEK-293T cells were plated on cover slips prior to treatment with a control siRNA or siRNAs against *FMR4*. At 72 hours post transfection, the cells were fixed with 4% paraformaldehyde (pH 7.4) for 15 min at room temperature, followed by permeabilization with 0.1% triton-X in PBS. **T**dT- mediated d**U**TP **n**ick **e**nd **l**abeling (TUNEL) reaction mixture was added to the cells and incubated at 37C for 1 hour (Roche, Indianapolis, IN). The cells were then stained with DAPI and images were captured using a confocal microscope.

## Supporting Information

Figure S1 Northern blot analysis of *FMR1* and *FMR4* in human adult tissues. *FMR1* and *FMR4* are co-expressed in some but not all of the human adult tissues examined.
(0.38 MB TIF)

Figure S2 Bioinformatic analysis of the genomic DNA sequence encompassing *FMR4*.
(0.17 MB DOC)

## References

[1] Carninci P, Kasukawa T, Katayama S, Gough J, Frith MC (2005). The transcriptional landscape of the mammalian genome.. Science.

[2] Cheng J, Kapranov P, Drenkow J, Dike S, Brubaker S (2005). Transcriptional maps of 10 human chromosomes at 5-nucleotide resolution.. Science.

[3] Birney E, Stamatoyannopoulos JA, Dutta A, Guigo R, Gingeras TR (2007). Identification and analysis of functional elements in 1% of the human genome by the ENCODE pilot project.. Nature.

[4] Bartel DP (2004). MicroRNAs: genomics, biogenesis, mechanism, and function.. Cell.

[5] Fire A, Xu S, Montgomery MK, Kostas SA, Driver SE (1998). Potent and specific genetic interference by double-stranded RNA in Caenorhabditis elegans.. Nature.

[6] Carmell MA, Girard A, van de Kant HJ, Bourc'his D, Bestor TH (2007). MIWI2 is essential for spermatogenesis and repression of transposons in the mouse male germline.. Dev Cell.

[7] Houwing S, Kamminga LM, Berezikov E, Cronembold D, Girard A (2007). A role for Piwi and piRNAs in germ cell maintenance and transposon silencing in Zebrafish.. Cell.

[8] Katayama S, Tomaru Y, Kasukawa T, Waki K, Nakanishi M (2005). Antisense transcription in the mammalian transcriptome.. Science.

[9] Pang KC, Frith MC, Mattick JS (2006). Rapid evolution of noncoding RNAs: lack of conservation does not mean lack of function.. Trends Genet.

[10] Ponjavic J, Ponting CP, Lunter G (2007). Functionality or transcriptional noise? Evidence for selection within long noncoding RNAs.. Genome Res.

[11] Wahlestedt C (2006). Natural antisense and noncoding RNA transcripts as potential drug targets.. Drug Discov Today.

[12] Migeon BR (2002). X chromosome inactivation: theme and variations.. Cytogenet Genome Res.

[13] Rinn JL, Kertesz M, Wang JK, Squazzo SL, Xu X (2007). Functional demarcation of active and silent chromatin domains in human HOX loci by noncoding RNAs.. Cell.

[14] He L, Thomson JM, Hemann MT, Hernando-Monge E, Mu D (2005). A microRNA polycistron as a potential human oncogene.. Nature.

[15] Ikeda S, Kong SW, Lu J, Bisping E, Zhang H (2007). Altered microRNA expression in human heart disease.. Physiol Genomics.

[16] Mehler MF, Mattick JS (2007). Noncoding RNAs and RNA editing in brain development, functional diversification, and neurological disease.. Physiol Rev.

[17] Verkerk AJ, Pieretti M, Sutcliffe JS, Fu YH, Kuhl DP (1991). Identification of a gene (FMR-1) containing a CGG repeat coincident with a breakpoint cluster region exhibiting length variation in fragile X syndrome.. Cell.

[18] Garber K, Smith KT, Reines D, Warren ST (2006). Transcription, translation and fragile X syndrome.. Curr Opin Genet Dev.

[19] Oostra BA, Chiurazzi P (2001). The fragile X gene and its function.. Clin Genet.

[20] Hinds HL, Ashley CT, Sutcliffe JS, Nelson DL, Warren ST (1993). Tissue specific expression of FMR-1 provides evidence for a functional role in fragile X syndrome.. Nat Genet.

[21] Tassone F, Hagerman RJ, Taylor AK, Gane LW, Godfrey TE (2000). Elevated levels of FMR1 mRNA in carrier males: a new mechanism of involvement in the fragile-X syndrome.. Am J Hum Genet.

[22] Oostra BA, Willemsen R (2003). A fragile balance: FMR1 expression levels.. Hum Mol Genet.

[23] Engstrom PG, Suzuki H, Ninomiya N, Akalin A, Sessa L (2006). Complex Loci in human and mouse genomes.. PLoS Genet.

[24] Sreeram N, Wren C, Bhate M, Robertson P, Hunter S (1989). Cardiac abnormalities in the fragile X syndrome.. Br Heart J.

[25] Tassone F, Hagerman RJ, Taylor AK, Hagerman PJ (2001). A majority of fragile X males with methylated, full mutation alleles have significant levels of FMR1 messenger RNA.. J Med Genet.

[26] Prasanth KV, Prasanth SG, Xuan Z, Hearn S, Freier SM (2005). Regulating gene expression through RNA nuclear retention.. Cell.

[27] Scacheri PC, Rozenblatt-Rosen O, Caplen NJ, Wolfsberg TG, Umayam L (2004). Short interfering RNAs can induce unexpected and divergent changes in the levels of untargeted proteins in mammalian cells.. Proc Natl Acad Sci U S A.

[28] Waldstein G, Hagerman R (1988). Aortic hypoplasia and cardiac valvular abnormalities in a boy with fragile X syndrome.. Am J Med Genet.

[29] Chureau C, Prissette M, Bourdet A, Barbe V, Cattolico L (2002). Comparative sequence analysis of the X-inactivation center region in mouse, human, and bovine.. Genome Res.

[30] Nesterova TB, Slobodyanyuk SY, Elisaphenko EA, Shevchenko AI, Johnston C (2001). Characterization of the genomic Xist locus in rodents reveals conservation of overall gene structure and tandem repeats but rapid evolution of unique sequence.. Genome Res.

[31] Davidow LS, Breen M, Duke SE, Samollow PB, McCarrey JR (2007). The search for a marsupial XIC reveals a break with vertebrate synteny.. Chromosome Res.

[32] Hore TA, Koina E, Wakefield MJ, Marshall Graves JA (2007). The region homologous to the X-chromosome inactivation centre has been disrupted in marsupial and monotreme mammals.. Chromosome Res.

[33] Okamoto I, Otte AP, Allis CD, Reinberg D, Heard E (2004). Epigenetic dynamics of imprinted X inactivation during early mouse development.. Science.

[34] Ladd PD, Smith LE, Rabaia NA, Moore JM, Georges SA (2007). An Antisense Transcript Spanning the CGG Repeat Region of FMR1 is Upregulated in Premutation Carriers but Silenced in Full Mutation Individuals.. Hum Mol Genet.

