# Supplementary figures and images for: A Novel RNA Transcript with Antiapoptotic Function Is Silenced in Fragile X Syndrome
Source: PLoS One. 2008 Jan 23;3(1):e1486. doi: 10.1371/journal.pone.0001486 (PMC2194623; doi:10.1371/journal.pone.0001486)

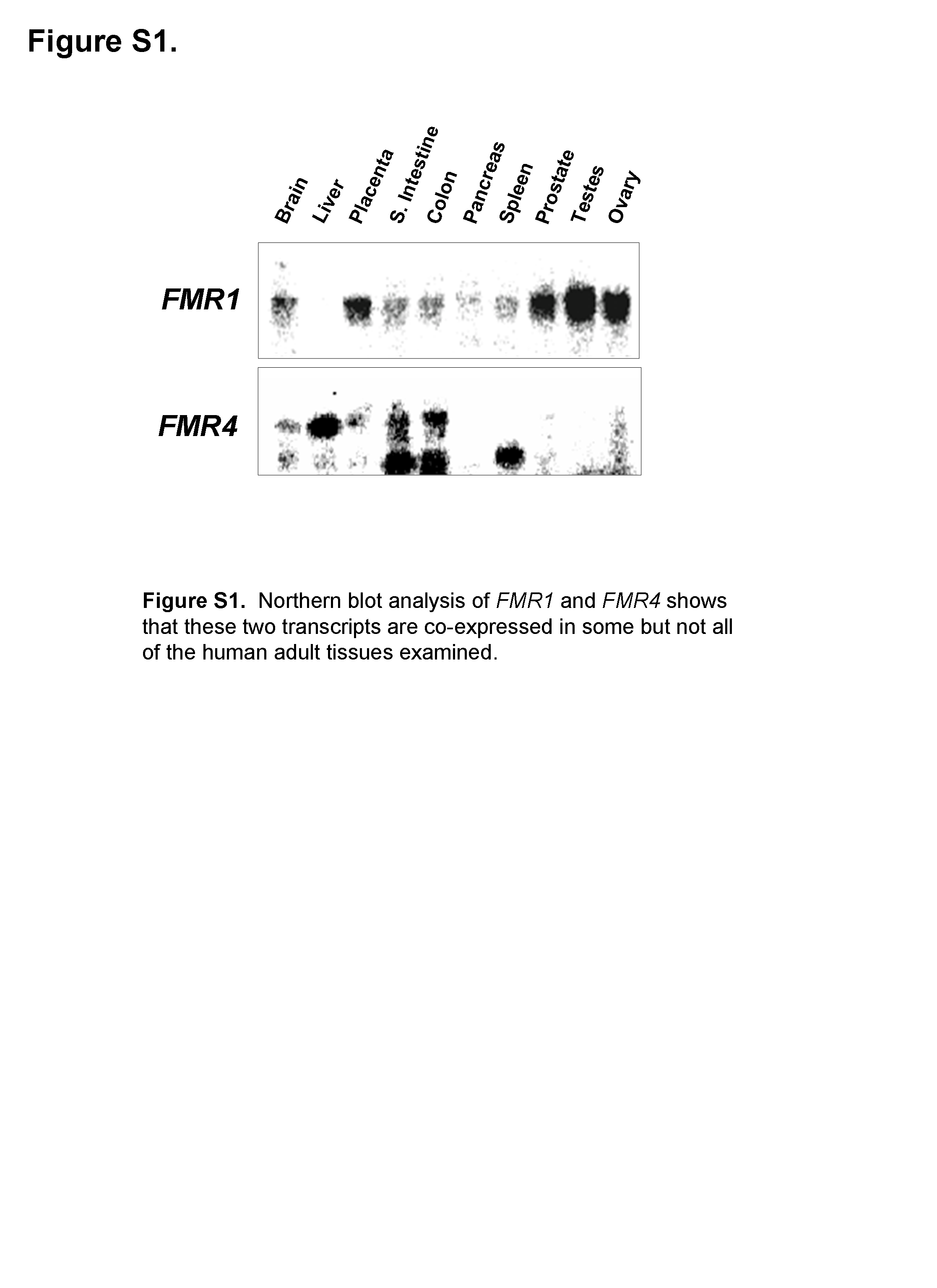

Supplement: Figure S1 — Northern blot analysis of FMR1 and FMR4 in human adult tissues. FMR1 and FMR4 are co-expressed in some but not all of the human adult tissues examined. (0.38 MB TIF) [file pone.0001486.s001.tif]
