# Supplementary material for: A Novel RNA Transcript with Antiapoptotic Function Is Silenced in Fragile X Syndrome
Source: PLoS One. 2008 Jan 23;3(1):e1486. doi: 10.1371/journal.pone.0001486 (PMC2194623; doi:10.1371/journal.pone.0001486)
Supplement: Figure S2 — Bioinformatic analysis of the genomic DNA sequence encompassing FMR4. (0.17 MB DOC) [file pone.0001486.s002.doc]

```

                  *        20         *        40         *        60
Human  : --------------------AATTATTCTAGT-ACACTAACCATCATAGTAGAGGTATT- :   38
Chimp  : --------------------AATTATTCTAGT-ACACTAACCATCATAGTAGAGGTATT- :   38
Rhesus : --------------------AATTATTCTGGT-ACACTAACCATCACAGTAGAGGTATT- :   38
Cow    : --------------------AGTGATGATAGT-ACATTAAGCATCCTAGGTGAGGTATT- :   38
Dog    : --------------------AGTGATTGTAGT-ACATTAAGCATCATAGGAGAGGTATC- :   38
Cat    : --------------------AGTGATCATACT-ACATCAAGCATCCCAGGAGAGGTATTT :   39
Horse  : --------------------AGTGATTCCAAT-ACAGTAAGCGTCATAGAAGAGGTATT- :   38
Mouse  : --------------------AATAATTCTACT-GTATTGTAAATGACAGTA-AAATATT- :   37
Rat    : ATGTAGCCTTTGACCACATTAGAGATTCTTCCAGCAATATGGTGGGCAGAA-ATACTTT- :   58
                             A t ATt ta t acA taa catc  AG agAggtaTt

                  *        80         *       100         *       120
Human  : CAACATTTGTTGAGTCTCTGCTATATGCCAAGCAGTGTAATGAGGAAGCAGAGGGTATGC :   98
Chimp  : CAACATTTGTTGAGTCTCTGCTATATGCCAAGCAGTGTAATGAGGAAGCAGAGGGTATGC :   98
Rhesus : CAACATTTGTTGAGTCCCTGCTATATGCCAAGCAGTTTA--------------GGTATGC :   84
Cow    : CAACATCAACTGAGTCCCTTCTATATGCTGAGTTGTGCAGTGAGAAA-CAGGAGGCATGT :   97
Dog    : CAACATTTACTGACTCCCTGCCATATGCCAAGCAGTGTGGTGACGAAGCAGGACGTGTGT :   98
Cat    : CAACATTTACCGAATCCCTGCGATGTGCCAAGCAGTGTGGTGAGGAAGCAGGAGATATGT :   99
Horse  : CAACATTTACTGAGGCCCTGCTATATGCTAAACAGTGTGGTGAGGAAGCAGGAGGTACGT :   98
Mouse  : --AAGC--ATTCAGAGCCTGTTTTATAATCAG-TGCAAAAGAAGAAACAGGAAGT---GC :   89
Rat    : --AAAATGATTGCTACAGGACTTCAAGACCAG-GGGATTTTACTCTTTCTTCCCTCAAAC :  115
         caAcatt   tga  c ctgctatatgc  Ag  Gt t  t a  aa c g  g  a g

                  *       140         *       160         *       180
Human  : A-----CAAAGTTCTACAAGAGCACA--------AAATAAGTTCTGGCAAAGGTTTGTAA :  145
Chimp  : A-----CAAAGTTCTACAAGAGCACA--------AAATAAGTTCTGGCAAAGGTTTGTAA :  145
Rhesus : A-----CAAAGTTCTACAAGAGCACA--------AAATAAGTTCTGGCAAAGGTTTGTAA :  131
Cow    : A-----CAAAGT-CTTTAAGAGCACA--------AAAGTGGTTCTTGCAAAGGTTCGTAA :  143
Dog    : A-----CAAAGTTCTATGAGAGCACA-----CACAAAAAAATTC------------GTAA :  136
Cat    : A-----CAAAGTTCTGCGAGAGCACAGA---CACACACAAATTCTTGCAGACGTTCGTAC :  151
Horse  : AACGTACAAAGTTTC-TAAGAGCACA--------AAAGAAATTCGTGCAAAGGTTTGTAA :  149
Mouse  : A---TACACAACTCTGTAAGAGCACACT------CGCAAAATTCTTGCCACAGTGCATGA :  140
Rat    : A---GCTATAGCGAGGAGAGAGACTATTTCCTCACACAAAATTCTTGCCATGGTACATGA :  172
         A     cAaAgttct   AGAGcacA        aaa aa TTCt gc a  gt  gTaa

                  *       200         *       220         *       240
Human  : AGACATTCACAAGGGTTTTCACCACAGTATGACTTCAGGGAGTTGGCAGTAACCTAGATG :  205
Chimp  : AGACATTCACAAGGGTTTTCACCACAGTATGACTTCAGGGAGTTGGCAGTAACCTAGATG :  205
Rhesus : AGACATTCACAAGGGTTTTCACCACAGTATGACCTCAGGGAGTTGGCAGTAACCTAGATG :  191
Cow    : AGACACACACAAGGATTTTCACCACAGAGTGACATAAAGGAGTTGGCAGTATCCTAGACG :  203
Dog    : AGACATTCACAAGGGTTGTCACCACAGTGTGACATCAGGGAGTTGGCAGTAGCCTAGAAG :  196
Cat    : ACACATTCACAAGGGTTTTCACCACAGTGTGACAGCAGGGAGTCGGCAGTAACCAAGATG :  211
Horse  : AGACATTCACAAGCGTTTTCACCACAGTGTGACATCAGGGAGTTGCCAGTAACCTAGACG :  209
Mouse  : AGAAGAC-ACAAGAGTTTTCGTGACCCTGGGAATGGTGGCATATGCCTTTATTCCCAGTA :  199
Rat    : AGACAATTGCAAGAGTTTTCATGATCCCGGGAATGGTGGCACATGCCTTTATTCCCAGTA :  232
         AgAca tcaCAAG gTTtTCaccAcagt tGAc   agGgAgttG CagTA cC aga g

                  *       260         *       280         *       300
Human  : CCCGATCAGTAGGGATATGTATG---AATAAAATTTCTGGCATACTCGGTAGCA------ :  256
Chimp  : CCCGATCAGTAGGGATATGTATG---AATAAAATTTCTGGCATACTCGGTAGCA------ :  256
Rhesus : CCCGATCAGTAGGGATATGTATG---AATACAATTTCTGGCATACTCAGTAGCA------ :  242
Cow    : CCC-ATCACTAGGGAAAT------------------------------------------ :  220
Dog    : CCC-ATCACTAGGAAAATGTATCATCAGTAAAGTCTATGGAATACTCAGCAGCAGTGAGA :  255
Cat    : CCC-GGCACTAGGGAAATGTATC---AGTAAAATCTATGGAATACTCAGCAGCAGGAAGA :  267
Horse  : CCC-ATCACTAGGGAAATGTATC---AATACAATTTATGGAATACTCAGCAGCAGTTAGA :  265
Mouse  : CTTTGGTAGCAGGAGCAAATAGAT----CACCATGTGTTCAAGGCCTGCCAAGG------ :  249
Rat    : GCTAGGTGACAGACGGAAACAGAT----CACCATGTGTTCAAGGCCCGTCGAGG------ :  282
         ccc   ca tAGg a At ta        a  at t t   a  c c   a

                  *       320         *       340         *       360
Human  : -------AACTAGGTGTACACACAGCAATGTGGGTATAGCTC-AAAAACAGACTGTTGAG :  308
Chimp  : -------AACTAGGTGTACACACAGCAATGTGGGTATAGCTC-AAAAACAGAGTGTTGAG :  308
Rhesus : -------AACTAGGTGTACACACAGCAATGTGGGTATAGCTC-AAAAACAGAGTGTTGAG :  294
Cow    : --------------------TGCAGGAATGTGGATATAGCTC-AAGAACGTAGCATTGAG :  259
Dog    : AGCCAAAAACTAGGTGTACACACAGCAACGTGGATATAACTC-AAGAACACAGTATTGAG :  314
Cat    : AGCAAAAAACTAGGTGTACACACAGCAATGTGGATATAATTC-GAGAACACAGGGTTGAG :  326
Horse  : AGCCAAAAACTAGGTGTACACACAGCAACGTGGATATAGCTC-AAGAACGTGGTGCTGAA :  324
Mouse  : ---------ATAAATACTGAGACACTGTCCCAAAAGCAATTTTAAAAATACAGAT-TTAA :  299
Rat    : ---------CTGAATACTAAGACATTGTCTCAAAATAAACTTTAAAAGTAAAAAAATTAA :  333
                  cta  t    a aCAg aa gtgg tatA cTc aA Aaca ag   TgA

                  *       380         *       400         *       420
Human  : T-AAAACAGTGGGAAATA-GAGATTTACAGTCCAATACCATCTCTGTAAATGCAAGAGGC :  366
Chimp  : T-AAAACAGTGGGAAATA-GAGATTTACAGTCCAATACCATCTCTGTAAATGCAAGAGGC :  366
Rhesus : T-AAAACAGTTGTAAAGA-GAGATTTACAGTCCAATACCATCTCTGTAAACGCAAGAAGC :  352
Cow    : TGAAAACAGTGGGAAATAAGAGATTTACAGTCCAGGACCATCTTCATAAGTGTAAAAGGC :  319
Dog    : TGAAAACAGTGAGAAACA---CATTTCCAGTCCAATATCATCTCTTTAAATGTAAA-GGC :  370
Cat    : CGAAAGCAGTAAGAAAAA---CGTTTCCAGTCCAATACCATCTCTGTAAATGTAAA-AGC :  382
Horse  : TGAAAACAGTGGGAAACA---GATTCACAGTCCAATACCACCTCTGTAAATGCAAAAGGC :  381
Mouse  : TGGTAGCAGTGAGAAACA---GATTTACAGTCCAGTACCACGTCTGTGTATGCAAAAGCC :  356
Rat    : TGGTAGTAATGAGAAGCA---GATGTGTAGTCCAGTAGCATCTCTGCACATGCAAAAGCC :  390
         t aaA cAgTg gAAa A   gaTtt cAGTCCA tAcCAtcTctgtaaatG AA aggC

                  *       440         *       460         *       480
Human  : ATAAACAAAACATTATCTGTGTT---AAATTATCAAGGATCTCTATCGAACATATT-GCA :  422
Chimp  : ATAAACAAAACATTATCTGTGTT---AAATTATCAAGGATCTCTATCGAACATATT-GCA :  422
Rhesus : ATAAACAAAACATTGTCT----T---AAATTATCAAGAATCTCTATCAAACACATT-GCA :  404
Cow    : ATGCACGAAACCCCATGTC--TT---AAATGATCAAGAATCCATACCAAACATATT-GCA :  373
Dog    : ATACACAAAACACTGCAAGTCTT---AAATTATCAAGGATCTAGATCAA-CACATC-GCA :  425
Cat    : ATACACAAAACACTGCAGGTCTT---AAACTATCAAGGAACTATACCAA-CACGTT-GCA :  437
Horse  : ATACGCAAAACACTGCATGTCTT---AAACTATCAAGGAGCTACATCAAACAGATT-TCA :  437
Mouse  : ATACAC-ATATA--------------GCACACTAGAAGTAAAG----------------- :  384
Rat    : ATGCAC-ATATACTATTTATCTTGTGGCTCACTTGATGGGACATGTCTTTTATAACAGCA :  449
         ATa aC AaAca t       tt   aaa  aTcaAgga c   a c a ca at  gca

                  *       500         *       520         *       540
Human  : GCTTGTGTCTAGAAGA---ATGAGAGTGGGGATCGAGAAAGATGAGGAAAAAATAATATA :  479
Chimp  : GCTTGTGTCTAGAAGA---ATGAGAGTGGGGATCGAGAAAGATGAGGAAAAAATAATATA :  479
Rhesus : GTTTGTGTCTAGAAGA---ATGAG--TGGGGATTGAGGAAGATGAGGAAAAAATAATATA :  459
Cow    : GTTTGTATCTAGAAGATGAATGGGAGTGGGTATTGAGAAAGACAAGGACAAA-TAATTTA :  432
Dog    : GTTGGTATCTAGAAGAAGGATGAGAGTAG-TATT----AAGATAATGAAAAA-TAATAA- :  478
Cat    : GTTGGTATCTAGAGGCAGAATGAAAGTGGGTATT----AAGATGGGGGAAAA-TAATA-- :  490
Horse  : G---GTGTCTAGAAAGAGAATGAGAGTGGGGATTGAGAAAGACGAGGAAAAA-TAATATA :  493
Mouse  : -----AAAGAACAGGGAATTCAAGGATAGCCTCTACTAATTCTGAGTTGTGGCCAACCTG :  439
Rat    : CTCTAGAAGAATAGGGAACTCAAAGATAGGCTGTACTAATTGTGAGTTCTGGCCACCCTG :  509
         g   gt tctAgA g    atgag  T Gg att    Aagatgagg  aaa tAat t

                  *       560         *       580         *       600
Human  : AACACTATAAAATAATGTAAACAAGGA-----CCCTGTAGGGACTGAT-ATGACAATGTG :  533
Chimp  : AACACTATAAAATAATGTAAACAAGGA-----CCTTGTAGGGACTGGT-ATGACAATGTG :  533
Rhesus : AAAAATGTAAAATAATATAAACAAGGA-----CCTTGTAGGGACTGAT-ATGACAATGTG :  513
Cow    : ACCAAAGTAAAACAATATAAATAAGAAAAGGGCCTTGCAGGGACTGATGATGACAACGTG :  492
Dog    : --CAACATAAAATAATAAAAATAAGAAGGG---TTGGCAGTGACTGATGATGCTAATGTG :  533
Cat    : --TAAAATAATGTAAATGGGACAGG--GGC---CTGGCAGGGACTGATGATGATAATGTG :  543
Horse  : AACAATATAAAATAATACAAATAAGAGGGGG-TCTTGCAGGGACCGGTGATGGCAATGTG :  552
Mouse  : --CCCTACAAAAAAATCTGTCCTTAAAAACA--CTATCTTAAACTATTAATGATA---TG :  492
Rat    : --CCATACAAGAAAATCTGTCCTTAAAAACA--CTATCTTAAACTATTAATGATA---TG :  562
           ca  atAAaa AAt    a a g a      ct g ag gACtg T ATGa Aa gTG

                  *       620         *       640         *       660
Human  : CTGAAAAT---------------------------------TGAGGAGCAAAGTTAACT- :  559
Chimp  : CTGAAAAT---------------------------------TGAGGAGCAAAGTTAACT- :  559
Rhesus : CTGAATAT---------------------------------TGAAGAGCAAAGTTAACT- :  539
Cow    : CTGAGAAT---------------------------------TGTGGAGCAGAGTAAACT- :  518
Dog    : CTAAGAATCGAGGAGCAAAGTTATTTTTTTTTGGTTGTTTTTGAGGAGCAAAGTTAACTC :  593
Cat    : CTGCGAAT---------------------------------TGAGGAGCAAAGTTAACTG :  570
Horse  : CTGAGAAT---------------------------------TGAGGAGCAAAGTTAACTC :  579
Mouse  : TCAAACATTA----------CAACTGTTTTCTAGTAGAATGAGACAAGTAATATACACAA :  542
Rat    : TCAAACATTA----------CAACTGGTTTCTAGAAGAATGAGACCAATAACAAATACAA :  612
         ct a  AT                                 tGa gAgcAaagt aACt

                  *       680         *       700         *       720
Human  : ----CTCTGTACCTGAGATA-AAATAACTAGCTAATAGGAATCCAGC------------- :  601
Chimp  : ----CTCTGTACCTGAGATA-AAATAACTAGCTAATAGGAATCCAGC------------- :  601
Rhesus : ----CTCTGTACCTGAGAGA-AGATAACTAGCTAATGGGAATCCAGC------------- :  581
Cow    : ----CTCTGCACCTGAGATACAAATAACAAGCTAATGGGAATCCAGC------------- :  561
Dog    : AACTCTCTGCACCTGAGG-AAAAATAACTAGCTAATGGGAATCCATCCGAGGGGCACCTG :  652
Cat    : AATTCCCTGCACCTGAGG-TAAAATAACTGGCTAATAGGAATCCATC------------- :  616
Horse  : AACTCTCTGCACCTGAAGTAAAAACAACGCGCTAATAGGAACCCAGC------------- :  626
Mouse  : TAATATGAGCAAAAGAGGGGCCATGTAAGAACTAATGGTGAGCATGCTGAGAATTGAAAT :  602
Rat    : TAATATGAGCAAA-GAGGGGCCATGTAAGAACTAATGGTGAGCATGCTGAGAATTGAAAG :  671
             ctctG AcctGAg    aaa aAc agCTAAT GgaA CcagC

                  *       740         *       760         *       780
Human  : ------------------------------------------------------------ :    -
Chimp  : ------------------------------------------------------------ :    -
Rhesus : ------------------------------------------------------------ :    -
Cow    : ------------------------------------------------------------ :    -
Dog    : AATGGCTCAGTCAGCATCCAACTCCGGATTTGGGTCCAGGTCATGATCTCAGGGTCCGGG :  712
Cat    : ------------------------------------------------------------ :    -
Horse  : ------------------------------------------------------------ :    -
Mouse  : AAATGCTTACTGAAATATTTGCAACTGAAGTGAAATAATTACACAATCGGACT--CTACC :  660
Rat    : AAATGCTCACTCAAACATGTGTACCTGAAGTGAAATAACTACACAATAGGAAAAACTACC :  731


                  *       800         *       820         *       840
Human  : ------------------------------------------------------------ :    -
Chimp  : ------------------------------------------------------------ :    -
Rhesus : ------------------------------------------------------------ :    -
Cow    : ------------------------------------------------------------ :    -
Dog    : GATGGACCCCCCAGTCAGGTTTTCCACTAAGTGGCCATTGGGTTGGGGATTTTTTTTTTC :  772
Cat    : ------------------------------------------------------------ :    -
Horse  : ------------------------------------------------------------ :    -
Mouse  : TAATAACTTTAAGGGGGAAAGAAAGTTATGCACAGTGTCATGACATCATGAAACTATGTC :  720
Rat    : TAATAACTTTAAAGGG-AAAGAAAATCATGAACATCATGA--AAATCATGAAACTATGTC :  788


                  *       860         *       880         *       900
Human  : ------------------------------------------------------------ :    -
Chimp  : ------------------------------------------------------------ :    -
Rhesus : ------------------------------------------------------------ :    -
Cow    : ------------------------------------------------------------ :    -
Dog    : CTTTTCCTTTGGCCCTTGCCTGGTTTTTTTTTTTTAAAAAAATTTTTTTTTCCTTCCATC :  832
Cat    : ------------------------------------------------------------ :    -
Horse  : ------------------------------------------------------------ :    -
Mouse  : TTAGGACAGTAACCTTTGCGAATTACTGATATGTTTGAAAGCCCTGAGAAAACTAC---- :  776
Rat    : TAAGGATAGTGAC-TTTGTGAAGTACTGAAATGTTTGAAAGCCCTGAG------------ :  835


                  *       920         *       940         *       960
Human  : ------------------------------------------------------------ :    -
Chimp  : ------------------------------------------------------------ :    -
Rhesus : ------------------------------------------------------------ :    -
Cow    : ------------------------------------------------------------ :    -
Dog    : CAGGAGAACCCCCGGGGGGGGGGGGGGGGCGGGGAGACAGAGACACAGGCAGGCTCCATG :  892
Cat    : ------------------------------------------------------------ :    -
Horse  : ------------------------------------------------------------ :    -
Mouse  : ----------------------------------ATATCTTACACTTGGAAGATAGAATC :  802
Rat    : ------------------------------------------------------------ :    -


                  *       980         *      1000         *      1020
Human  : ------------------------------------------------------------ :    -
Chimp  : ------------------------------------------------------------ :    -
Rhesus : ------------------------------------------------------------ :    -
Cow    : ------------------------------------------------------------ :    -
Dog    : CAGGAAGCCTGATGTACTGGATTCTGGTCTCTAAGATCAGGCCCTGGGCTGAAGGTGGTG :  952
Cat    : ------------------------------------------------------------ :    -
Horse  : ------------------------------------------------------------ :    -
Mouse  : TTCTCAATCAATATGTCCCCTTTTATATCACTGGAGTTAAAGCTAATGTTATGCCCCATC :  862
Rat    : CTTTCAA---------------------------AGTTAAAGCTAATGTTATGCCCC--C :  866


                  *      1040         *      1060         *      1080
Human  : -----------------------------------------------------------T :  602
Chimp  : -----------------------------------------------------------T :  602
Rhesus : -----------------------------------------------------------T :  582
Cow    : -----------------------------------------------------------C :  562
Dog    : CTAAACTGCTGAGCCACCTAGGCTCCCCTAAAATAAATCTTTTAAAAACGGAATCCATCT : 1012
Cat    : -----------------------------------------------------------T :  617
Horse  : -----------------------------------------------------------T :  627
Mouse  : TGGTCTATGGTGAGATTTGTATTCAACCCTACAGGATTCTTAATATATTGTGGCTACTCC :  922
Rat    : TAGTCTATGGTGAGATTTGTAGTGGGCCCTACTGGATTCTTAATATGTTGTGGCTACTCC :  926


                  *      1100         *      1120         *      1140
Human  : GAAAACCTTAAGGTGCAGGGC-CTCTATGGGGCCCAGGAAGGATGTGTA--GAGACATGA :  659
Chimp  : GAAAACCTTAAGGTGCAGGGC-CTCTATGGGGCCCAGGAAGGATGTGTA--GAGACATGA :  659
Rhesus : GAAAACCTTAAGGGGTAGGGC-CTCTACGGGGCCCAGGAAGGATATGTA--GAGACATGA :  639
Cow    : GATAACCCTAATGAGAAGA----TTTCTGGGGGCCAAGAAGCACTGG----GATACATGA :  614
Dog    : GATAACCCTAAGGAGAAGGGT-AATCCTGGGGATA---ATCCCTGTGTGTAGAGACATGG : 1068
Cat    : GATAACCCTAAGGAGAAGGGT-GGTTCTGGGAAAGCAGAAGTGTGTGAGTAGAGACATGG :  676
Horse  : GATGACCCTAA--AGAAACGT-ATTTCTGGGGACCAGGAAGCATGTGTG--GAGACATGA :  682
Mouse  : AAAGATCCTAGTTCAATGGGTCATTTCTGTTTACCATAGCCTCAAGGAATGTGGCCCTTG :  982
Rat    : AAAGACCCTAGTTCAATGGGTCATTTCTGTTTACTATAGCCTCAAGGAATATGGCCCTTG :  986
         gA  AcC TAa   g aggg   t t tGgg  c a  a       G    gagaCaTg

                  *      1160         *      1180         *      1200
Human  : ACGGATGAAAGTGCATCACAGGTTCAGGGAACAACAC-AGGTTGAGTGTGGCTTGTAGTA :  718
Chimp  : ACGGATGAAAGTGCATCACAGGTTCAGGGAACAACAC-AGGTTGAGTGTGGCTTGTAGTA :  718
Rhesus : ACGGATGAAAGTGCATCACAGGTTCAGGGAACAACAC-AGGTTGAGTGTGGCTTGTAGTA :  698
Cow    : AGGCACGAAAGTGCATCAGAGCTTCAGGGAACAGCACCAGGTTTAGTGTTGGTTGCAGT- :  673
Dog    : AGGCATGA-AGTGACTCATAGATTCACGGAACAGCACCAGGTTTAGCAGGGCTTGTAGAA : 1127
Cat    : AGGCATGAGAGTGAATCATAGGTTCAGGGAACAGCACCAGGTTTAGCATGGCTTGTAGAG :  736
Horse  : AGGCATGAAAGTGCCTCACAGGTTCAGGGAATAGCACCAGGTTCAGTGTGGCTTGTACTG :  742
Mouse  : ATCCACTGCTTTAAAGCTGGAGTTGC---AACAACATTAATATTGTTCCGGCT-GAGGCA : 1038
Rat    : ATCCACTGTCTTAAAGCTGGAGTTCC---AAGTGCACTGAGATTGTCCTGGCT-GGGGCA : 1042
         A g A ga agTg atCa aggTTca ggAAca CAc aggtT ag  tgGcTtG ag

                  *      1220         *      1240         *      1260
Human  : AAAATGGTTGTGAAGAGTTGACA-TATTTTTAAGCCCTGGGTAAATTGAAC--AACAGCT :  775
Chimp  : AAAATGGTTGTGAAGAGTTGACA-TATTTTTAAGCCCTGGGTAAATTGAAC--A---GCT :  772
Rhesus : AAAATGGTTGTGAAGAGTTGACA-TATTTTTAAGTCCTGGATAAATTGAAC--AACATCT :  755
Cow    : -----GGTTATGAAGAGCTGATAATATTTGAATGCCTGGAGTAAACTGAAC--AACATCC :  726
Dog    : -----GTTT-TGAAGAGCTGATAATGTCTGAAAGCCCTGGATGAACTGAAC--AACACCC : 1179
Cat    : -----GTTTGTGAAGAGCCGGTAGCATTTGAAACTCCTGGGTGAACTGAATT-AACTCCC :  790
Horse  : -----GTTG-TGAAGAGCTGATAATCTTTGAAAGCCCTGGGTAAACTGAAC--AACACCC :  794
Mouse  : GGCTGGGAACTGGAAGCCTCACAT---CCTAG--CCCTCTACCATCTGCTTAGGACAACT : 1093
Rat    : GTCTGGGAACTGGAAGCCTAAAGTACTCTTTA--TCCTATGCCACCTGCCTGGGGCACCT : 1100
              G t  TGaAgag tga a   t t  a   Cctg  t Aa TGaa   aaca C

                  *      1280         *      1300         *      1320
Human  : TACACTTGGAGGGGTATAATCATTCTAATCAATGTGTCCCCTTTTACTATAATACATTGG :  835
Chimp  : TACACTTGGAGGGGTATAATCATTCTAATCAATGTGTCCCCTTTTACTATAATACATTGG :  832
Rhesus : TACACTTGGAGGGGTATAATCATTCTAATCAATGTGTACCCTTTTACTATAATATATTGG :  815
Cow    : TACACGTGGAAGT-TACAATCATCCGTATCAATCTGTCCCCCTTCTTTATATCGGGTTTA :  785
Dog    : TACACGTGGATGG-TATAACCATTCGAGTCAATCTGTCCCCTTTAACTACATTGGGGTTA : 1238
Cat    : TACACGTGCAGGG-TATGACCATTCTAATCAATCTGTCCCCTTTCACTACATCGGGGTGA :  849
Horse  : TACACGTGGAAGG-TATAATCATTCTAATAAATCTGTCCCCTTTTACTACACTGGGGTTA :  853
Mouse  : CACCCTTGCTCTTTCAAGTGCACACTTCCAAATAAGTCTTTCTT-GTCACATTCCAGTTG : 1152
Rat    : CACCCTTTGTCTTCCAAGTGCACACTTCCAAATAAGTCTTCTTT-GTGCCATTCCACCTG : 1159
         tACaC Tgga g  tA  a CAt Ct  t AAT tGTcccctTT   ta A t    t

                  *      1340         *      1360         *      1380
Human  : AGTTGCAGCTAATGCTCTGCTCCCAT---TCAGCCTATGATGAGATTCTCTTTCAGCCCT :  892
Chimp  : AGTTGCAGCTAATGCTCTGCTCCCAT---TCAGCCTATGATGAGATTCTCTTTCAGCCCT :  889
Rhesus : AGTTACAGCTAATGCTCTGCTCCCAT---TCAGCCT--GATGAGATTTTCTTTCAGCCCT :  870
Cow    : AGGCTGATGCTCTGCTCTGCTTCCAT---CCAGTCAACAGTGATAGTCTCTTTTAGCTGC :  842
Dog    : AAGAGCAG-----GCTCCGCTCCCAT---CCAGCCCGTGGTGAGATTCTCTTTCAGTCAC : 1290
Cat    : AAGAGCATA---TGCTCTGCCCCCGT---CCAGCC-----------------------GT :  880
Horse  : AAGATCAC-----GCTCTGCTCCCAC---CCAGCCTATCGTGAGGTTCCCCTTCAGTCCT :  905
Mouse  : GAATCATCTTGCTTCCGGGCTCACTTATCTCACACCACAGACAGGCTCATTTCCAACTGT : 1212
Rat    : GACTCATCTTGCTTCTGTCCTCACTTATCCCATGCCACAGACAGATTCATATCCAACTGT : 1219
         a     a     tgCtctgCtccC t    CAg C       ag  tc   t ca    t

                  *      1400         *      1420         *      1440
Human  : ATTGGG-TTCTTGGCCTCATGT-GACTACTCCAAAGACCCTAGTCCAAAAGGTCTTTCCT :  950
Chimp  : ATTGGG-GTCTTGGCCTCATGT-GACTACTCCAAAGACCCTAGTCCAAAAGGTCTTTCCT :  947
Rhesus : CTTGGG-GTCTTGGCCTCATGT-GACTACTTCAAAGACTCTAGTCCAAAAGGTCTTTCCT :  928
Cow    : ACTGGG-ATCTTTGCATCTTGT-GACGACTCCAGAAATCCTAGTTCAACAAATCACTCCT :  900
Dog    : CCTGCA-GTCTTCACAGCTTGT-GACTACCTCAGAAACCCTATTCCAATGGTTCATTTTT : 1348
Cat    : CCTGCG-GTCTTCACATCTTGT-GACTA--------------TTCCAATGGGTCACTCCT :  924
Horse  : ACTGGG-GTTTTTGCATCTTGT-GACTACTCCAGAAACCCTGGTCCAATGGGTCATTCCT :  963
Mouse  : AAAAGATATAACCACACTTTATAGACCACATCACAGTTGTGACTCCATATTTATAT-CTT : 1271
Rat    : GAAAGATATATTTACACTTTATAGACT---TCACAGTAGCAATTACAGATTTATATGTTT : 1276
           tgg   T tt  C  c TgT GACtac  ca a    c a TcCAa    tc ttc T

                  *      1460         *      1480         *      1500
Human  : GTTTGCTATGGCCTTGAGGAATGTGGCCCTAGATCCACCGCTTTAAAGCTGGAGTTCCAC : 1010
Chimp  : GTTTGCTATGGCCTTGAGGAATGTGGCCCTAGATCCACTGCTTTAAAGCTGGAGTTCCAC : 1007
Rhesus : GTTTACTATGGTCTTGAGGAATGTGGCCCTAGATCCACTGCTTTAAAGCTGGAGTTCCAC :  988
Cow    : GTTTA-TGTGGCCTCAAGGAATGTGGCCTT-GATCTGAGGCTTTCATGCTGGAGCTCCAC :  958
Dog    : GTGTACTGTGGCCTCAAGGAATGTGGCCTTTGATCCACTGCTTTAAAACTGGAGCCCCAC : 1408
Cat    : GTGTACTGTGGCCTCAAGGAATGTGGCCCTCGATCCGCTGCTGTAAAACTGGAGCTCCAC :  984
Horse  : GTTTACTGCGGCCTCAAGGAATGTGGCCCTTGATCCGCTGCTTTAAAATTGGAACTCCGT : 1023
Mouse  : CTTCA-TGCG---TGTATGTGTGTG--TA-GCATCCATGTGTGTGAGTATGGCCACACAT : 1324
Rat    : CTTCA-TGTG---TGTATGGGTGTA--TATGAATCCATGTGTGAGAGTATCCGCACACAA : 1330
         gTtta T tGg cT  AgGaaTGTggcc t gATCc   gcT t A   Tgga   cCa

                  *      1520         *      1540         *      1560
Human  : CAGCAGCAACAT------------------------------------------------ : 1022
Chimp  : CAGCAGCAACAT------------------------------------------------ : 1019
Rhesus : CAGCAGCAACAT------------------------------------------------ : 1000
Cow    : CAGAGGCAAGGCGGCTGCTCTGGCCAGGAGTGGGCTGGGAAGGGAACTGGAGGCCCAAGG : 1018
Dog    : CC---GTACTGCT-CTGGCTAGGCTAGGCGTAGGGTGGGAAGGAAAGTGGAGGCCCAGGC : 1464
Cat    : CCC-AGTGACACTGCTGCTCTGGCTAGGAGTGGGGTGGGAAGGGAACCGGAGGCCCAAGC : 1043
Horse  : CAGCAATAACACTGCTGCTTTGGCTAGGAGTGGGGTTGGAGGGGAACTGGAGGTCCACGG : 1083
Mouse  : ATGCCACAAAACATGTGTGGGGGTCACAGTACAACTTCT------AATGCAGTCCT---- : 1374
Rat    : ATGCCACAGGATATGTGTGGAGGTCGAAGGACAACTTCTTCC---AATGCAGTCCT---- : 1383
         c g    aa a

                  *      1580         *      1600         *      1620
Human  : ---CCTCTCATTCTGGGGCA-CCTGCCTGGGG----CAGGTCATCCTGCCTCTGCCAACT : 1074
Chimp  : ---CCTCTCATTCTGGGGCA-CCTGCCTGGGG----CAGGTCATCCTGCCTCTGCCAACT : 1071
Rhesus : ---CCTCTCATTCTGGGGCA-GCTGCCTGAGG----CAGGTCATCCTGCCTCGGCCAACG : 1052
Cow    : ATGCCTCGCATTCTGGGGCA-TCTGCCTGGGG----CAGTTCAACC-GCCTCCATCAACT : 1072
Dog    : ATACCTCTAATTCTGGGGCA-CCTGCCTGGGG----CGGTTCACCCAACCTCCACCA--- : 1516
Cat    : ATGCCTCTAATTCTGGGGCA-TCTGCCTGGGGG---CCGTTCACCCCACCTCCACCAGCT : 1099
Horse  : ATGCCTCACATTCTGGGGCG-CCTGCCTGGGG----CAGTTCACCCCGCCTCCAACAACT : 1138
Mouse  : -TGCCTTCTACCCAGAGTCTCTCTTTTTGTTGTTCGAAACTAGATAAGGCACACTGACCT : 1433
Rat    : -TGCCTTCTACCCAGAGACAGTCTTTTTGTTGTTCGAAGCTGGATAAGTCACACTGAACT : 1442
            CCTc  AttCtGgGgCa  CTgccTG gG    cag Tca cc gcCtC   cA ct

                  *      1640         *      1660         *      1680
Human  : CAGTGCTATTAGTTAACTCTCACCTGCCATAT-TCCAGCTGGAATC-------------- : 1119
Chimp  : CAGTGCTATTAGTTAACTCTCACCTGCCATAT-TCCAGCTGGAATC-------------- : 1116
Rhesus : CAGTGCTACCAGTTAACTCTCACCTGCCATAT-TCCAGCTGGAATC-------------- : 1097
Cow    : CAGTGCTGCCAGTTAACTCTCACTGGTCACAT-TCCAGGTGGAGTTT---CCCAGCCGTG : 1128
Dog    : ---------------ATTCTCTCTGGTCACAT-TCCAGCTGGAGTCACTTCCCAGTTCTG : 1560
Cat    : CAGGGCTGCCAGTTAACTCTCTCTGGTCACAT-TCTAGCTGGAGTCATTTCCCAGCCCTG : 1158
Horse  : CAGTGCTGCCAGTTAACTCTTACTGGTCACAT-TCCAGCTGGAGTCATTGCCCAGCTCTG : 1197
Mouse  : GTGAACTTCTGGGGAATCAACTTTGTGGGAGTGCTTATTTAGGCACTTAGGCCCAGCTTT : 1493
Rat    : GTGAACTTCTGGAGAATCAACTTTGGGGGAGTGATTATTTAGGCACTTAAGCCAAGCTTT : 1502
           g  ct    g  aA tctc c  g ca aT tc Ag TgGa tc

                  *      1700         *      1720         *      1740
Human  : ATCT-CCCCTTCTCCACCCCAGACTAGGTCATGTTCCGCCATCATGG-AAGCGCCTA-TT : 1176
Chimp  : ATCT-CCCCTTCTCCACCCCAGACTAGGTCATGTTCTGCCATCATGG-AAGCGCCTA-TT : 1173
Rhesus : ATTT-CCCCTTCTCCACCCCAGACTGGGTCATGTTCCGCCATCATGG-AAGCACCTA-TT : 1154
Cow    : ACCA-CCCTCCCCCCACCCCACACTGGGTCCTGCACCACTATCATAG-GTGCACCTA-TC : 1185
Dog    : ACCA-CCCCTGTCCCACCCCAGACTGGGTCCTGGCC-ACTATCATAG-GTGTATCTAATT : 1617
Cat    : AACAACCCCTCCCCCACCCCAGACTGGGTCCTGTCCCATTATTCTAG-GGGTATCTAGTT : 1217
Horse  : ACCA-CTCCTCCCCTACCCTAGACTGGGTCCTTTCCTACTATCATAG-GTGTACCTA-TT : 1254
Mouse  : CTATAGATTCTAGGAATTAGAAAATTCAGGATTTCATGCTCGCATAGCAAATGTTTTACC : 1553
Rat    : ATATGAATTCTAAGAATTCAAAAATTCAGAATTTCATGCTCGCATAGCAAATGCTTTACC : 1562
         a    c c     c Accc A AcT ggtc T t c  c atcaT G   g   cTa t

                  *      1760         *      1780         *      1800
Human  : CTTCATACCCCTTATCACAGCTGCAACTACTCATTTACTTGTCTGACAATTTGATTTATG : 1236
Chimp  : CTTCATACCCCTTATCACAGCTGCAACTACTCATTTACTTGTCTGACAATTTGATTTATG : 1233
Rhesus : CTTCATATCCCTTATCACAGCTGCAACTCCTCATTTACTTGTCTGATAATTTGATTTATG : 1214
Cow    : CTTCAGAGCCTTTATCACAGCTGCCACTCCAGATTTTCTTTTCTGATA-----ATGTATG : 1240
Dog    : CTTCACAGCCCTTATCTCAGTTGCAACTCCACATTTACTTTTCTGATAATTT-A----TG : 1672
Cat    : CTTCATAGCCCTTATCACAGTTGCAACTCGACATTTACTTTTCTGACAATTT-AGTTATG : 1276
Horse  : CATCATAGCCCTTATCACAGCTGCGACTCCACATTTACTTTTCTGGTAATTTGATTTATG : 1314
Mouse  : CCCT------TGAGCCACCTCTCCAGCTCCACACTTACGGTTTTGAGAAGTTGAATTTTG : 1607
Rat    : CACTACAG--CCAGCCACTTCTCCAGTCCCATATGTATAGTCTTGAGAGGTTGAATTTTG : 1620
         C tca a cccttatCaCagcTgCaactcc cAttTactt tcTGa Aa tt A tt TG

                  *      1820         *      1840         *      1860
Human  : TCCACCTA--CTTTGCTAGGTACTAAGTT-CAATGCTGGCAGTCGTTTCTTCTTTTTTTT : 1293
Chimp  : TCCATCTA--CTTTGCTAGGTACTAAGTT-TAATGCTGGCAGTCGTTTCTTCTTTTTTTT : 1290
Rhesus : TCCATCTA--CTTTGCTAGGTTCTAAGTT-CAATGCTGGCAGTCTTTTCTTCTTTTTTTT : 1271
Cow    : ATCATCTAGGTTCCTCTATGTTCCAAGTT-CAGTGAAGGTGGTCTTTTATTTTTTTTCAC : 1299
Dog    : TCCATCTA--TGCCTCTACGTTCCTAGGTGAAATGCTGGCAACTTTTCTCTTTTT----- : 1725
Cat    : TCCACCTA--TGCCTCTGGATTCCCAGTT-AAATGCTGGCAATCTTTTTTTTTTTTTTTT : 1333
Horse  : TCCGTCTA--CGTGTCTAGCATCCAAGTT-CAATGCTGGCAGTCTTGTCTTTCTTCATTT : 1371
Mouse  : CACATCTA--CTCCTGTAGGTTCCAATTT-CAATAGTGACAGTCTTTTTGTTGCTA---- : 1660
Rat    : CCCATCTA--CTCCTGTAGGTTCCAATGT-CAACAATGACAGTCTTTTTATTGCTA---- : 1673
          cCatCTA       cTaggttC aAgtT  Aatg tGgcagtctTtt  T  tT

                  *      1880         *      1900         *      1920
Human  : T--CTTTTCTGTTTTGCTCACCGATTTCTCGTTAGCACTTAGCACAGTGTCTGGCACAC- : 1350
Chimp  : T--CTTTTCTGTTTTGCTCACCGATTTCTCGTTAGCACTTAGCACAGTGTCTGGCACAC- : 1347
Rhesus : TTTCTTTTCTGTTTTGCTCACCGACTTCTCGCTAGCACTTAGCACAGTGCCTGGCACAT- : 1330
Cow    : T----TTTTTTTTTCTTTCACCAATGTTTCACTAGCACTCAGCAAGCCGTCTGGCACATA : 1355
Dog    : -----------------------ATTTCTCGCTAGCACTTAGCACAGTGCCTGGCATAT- : 1761
Cat    : C-------------TCTTCACCAATTTCTCACTAGTACTTATCACAATGCCCGGTATGT- : 1379
Horse  : T-------------TCTTCACCAATGTCTCACCAGCACTTTGCACAGTGCCTAGCATAT- : 1417
Mouse  : ---------------CCTCACCAAGGACTCACTAGTACTTAGGACAGTGCTTGGCACAT- : 1704
Rat    : ---------------CCTCACCAAGGATTCACTAGTACTTAGTATGGTGCTTGGCACAT- : 1717
                          tcacc A  tcTC ctAG ACTtagcAcagtG ctgGcA at

                  *      1940         *      1960         *      1980
Human  : ------------------------------------------------------------ :    -
Chimp  : ------------------------------------------------------------ :    -
Rhesus : ------------------------------------------------------------ :    -
Cow    : GTTGTTGTTTAGTCACTAAGCCGTGTCTGACTCTTTGAGACCCCATGGACTGCAGCCTGC : 1415
Dog    : ------------------------------------------------------------ :    -
Cat    : ------------------------------------------------------------ :    -
Horse  : ------------------------------------------------------------ :    -
Mouse  : ------------------------------------------------------------ :    -
Rat    : ------------------------------------------------------------ :    -


                  *      2000         *      2020         *      2040
Human  : ------------------------------------------------------------ :    -
Chimp  : ------------------------------------------------------------ :    -
Rhesus : ------------------------------------------------------------ :    -
Cow    : CATGCTGCAGGCTCCCCTATCCGTGGGATTTCCCAGGCAAGAATTGGAGCAGGTTGCCAT : 1475
Dog    : ------------------------------------------------------------ :    -
Cat    : ------------------------------------------------------------ :    -
Horse  : ------------------------------------------------------------ :    -
Mouse  : ------------------------------------------------------------ :    -
Rat    : ------------------------------------------------------------ :    -


                  *      2060         *      2080         *      2100
Human  : --------------------GATAGATGCTCCGTCAACTTCTCAGTT--------GGATA : 1382
Chimp  : --------------------GATAGATGCTCCGTAAACTTCTCAGTT--------GGATA : 1379
Rhesus : --------------------GATAGGTAC---ATAAACTTCTCAGTT--------GGGTA : 1359
Cow    : TTCCTTCTCCACTGGCATAGGGTAGAGGCTCAATAAACTTCTCAGCG--------GAATA : 1527
Dog    : --------------------AGCAGAGGCTCAATAAACGCCTCCTGTATCCTACGGAGTA : 1801
Cat    : --------------------GGTAGGGGCTCAATAAACTTCTCAGTT--------GGATA : 1411
Horse  : --------------------GGTAGGGGCTCAATAAGCTTCTCAGTT-------GGTTTA : 1450
Mouse  : --------------------GGTAGGCATTCAATGAACTTCTCAGTT--------GGATG : 1736
Rat    : --------------------GGTAGGCATTTAATGAACTTCTCAGTT--------GGATG : 1749
                             g tAG   ctc aT AaCttCTCagtt        G  Ta

                  *      2120         *      2140         *      2160
Human  : CCAGCATCCCG----AAGGGAACATGGATTAAGGCAGCTATAAGCACGGTGTAAAAACAG : 1438
Chimp  : CCAGCATCCCG----AAGGGAACATGGATTAAGGCAGCTATAAGCACGGTGTAAAAACAG : 1435
Rhesus : CCAGCATCCCG----AAGGGAACATGGATTAAGGCAGCTATAAGCACGGTGCAAAA-CAG : 1414
Cow    : CAAGCCTTCGA----AAGGAAACCGAGGTCGAGGTGAATACAAGCATGGTGCAGAAGCAA : 1583
Dog    : CAGGCCTTCCA----AAGGAAACCTAGGTCAACGTGGATGCAAGCTCGGTGCAGAAACAG : 1857
Cat    : AAAGCCTTCCA----AAAGAAACCTAGGTCAAAGTGGATACAAGGACAGTGCAGAAACAG : 1467
Horse  : CAAGCTTTCCA----AAGGAAACCTAAGTCAAGGTGGATACAAGAACAGTGCAGAAGCAC : 1506
Mouse  : CAAGCCTTCTGGATTAAAGGAGCATGGGTCAGGCTGGATATAAGCCCAATACAGAGGCCA : 1796
Rat    : CAAGCCTTCTGGATTAAGGGAGCATGGGTCAGGCTGGATAGGAGCCCAGTAGAGAGGCCA : 1809
         c aGC T C      AAgG AaC t g T aagg  g Ta aAGc c gTg A Aa Ca

                  *      2180         *      2200         *      2220
Human  : GAATAAGAAAAAGTTGAGG--------TTTGTTTCACAGTGGAA-------------TGT : 1477
Chimp  : GAATAAGAAAAAGTTGAGG--------TTTGTTTCACAGTGGAA-------------TGT : 1474
Rhesus : GAATAAGAAAAAGTTGAGG--------TTTGTTTCACAGTAGAA-------------GGT : 1453
Cow    : AAATGTGAAAAATTTAGGG--------TTTGTTTTACTGTGAGG-------------GGT : 1622
Dog    : GAATAAGAAAGAGCTGAGG--------TTTGTTGTACTGTGGGG------------GGGT : 1897
Cat    : GAATGAGAAAAAGCTGGGG--------TTTGTTGTACTGTGGGGTGGGGGGGGGGCGGGT : 1519
Horse  : AAAAGAGGAAGAGTTGCAG--------TTTGTTCTCCTGTGGGG------------AGTA : 1546
Mouse  : AAGGGGTGGGGAGGTGAT--------TTTGGTTCTGCTGTAGAA-------------GGC : 1835
Rat    : AGGGGGAGGAGGTGTGAAAGGCTAGACTTGGTTCTGCTGTGGAA-------------GGC : 1856
          aa   g aa ag Tg  g        TTtGTT   C GTgg               gg

                  *      2240         *      2260         *      2280
Human  : AA------AGGGTTGCAA-----GGAGGTGCATCGGCCCCTGTGGACAGGACGCATG-AC : 1525
Chimp  : AA------AGGGTTGCAA-----GGAGGTGCATCGGCCGCTGTAGACAGGACGCATG-AC : 1522
Rhesus : AA------AGGGTTGCAA-----GCAGGTGCATCGGCCCCTGTAGACAGGACGCATG-AC : 1501
Cow    : AAGCGGGGAGAGCTGCAA-----AGAGGTGCACTGGCCCCTGTAGATAAGTCACCTCCAG : 1677
Dog    : AAAGAGGGAGAGCTGCACCGAAAACAGGCGCACTGGCCCCTGCAGAGAGGTCACCTG-AC : 1956
Cat    : AAAAGGGGAGAGCTGCAA-GGAAACAGGTGCGCTGTCCCTGGTAGACAGGTCACCTG-AC : 1577
Horse  : AAACAGGGAGAGCTGCAA-GGAAACAGGTGAAG-GGCCCCTGTAGATACGTCCCCTG-GC : 1603
Mouse  : AAAGGCAGAGAGCTGCAT--GGAAAGGACCCAATGATGCACATAGACAGATCCCATG-AC : 1892
Rat    : ACAGGAAAAGAGCTGCAT--GGAGTGGACACAATGATGTGCATAGACAGGTTCCATG-AC : 1913
         Aa      AG G TGCA        aGg gca  G ccc  gtaGA Agg c C Tg ac

                  *      2300         *      2320         *      2340
Human  : TGCTACACACG--TGTTCACCCCACCCTCTGGCACAGGGTGCACATACAGTAGGGGC--- : 1580
Chimp  : TGCTACACACG--TGTTCACCCCACCCTCTGGCACAGGGTGCACATACAGTAGGGGC--- : 1577
Rhesus : TGCTACACACG--TGTTCACCCCACCCTCTGGTACAGGGTGCACATAGAGTAGGGGC--- : 1556
Cow    : TGCAAGGCGGG--CATGTGGTAGG---TGTGAAACAGTAGGGAAATTAACT--GTAA--- : 1727
Dog    : TGCTGTACACATGCGCACACTCCACCCTCCTATTCAGGGTGGGCATACAGTAGGGGCCAA : 2016
Cat    : TGCTGTACAAA--CGCCCACCCCGACGCCCTGTACAGGGTGGGTATACAGTAGGGGCAAA : 1635
Horse  : TGCTCTACACA--TGCCTACCCCAGCCTTTGGTACAGAGTGACTATACAGGAGGAGCGAG : 1661
Mouse  : TGCTTTACTGA--TGCCCTCCC-----TCTGGTACAGGGGGCATGTAGAAGAGGC----- : 1940
Rat    : TGCTTTCCTGA--TGCCCTCTC-----TCTGGTATAGGGTGCACATAGAAGAGGC----- : 1961
         TGCt  aC      g  c c c     tctgg acAGggtG   aTa A  agG

                  *      2360         *      2380         *      2400
Human  : -------AGAAATGAACCTCAAGTG--CTTAACACAATTTTTAAAAAATATAT------- : 1624
Chimp  : -------AGAAATGAACCTCAAGTG--CTTAACACAATTTTTAAAAAATATAT------- : 1621
Rhesus : -------AGAAATGAACCTCAAGTG--CTTAACCCAATTTTTAAAAAATATAT------- : 1600
Cow    : -------AGAAATCAAC---GAGTG--TTTAGCGCATTTTTTAAAACCTGT--------- : 1766
Dog    : ACAGTAGGAAAATAAATTGTAAAGAAATTTAACGAGTGTTTTAAGACATTTTTTTTTTTT : 2076
Cat    : ATAATAGGAAAATAAACTGTAAAGGAATT-AACAAGTGCTT-AAGACATTCTTTTTTTTT : 1693
Horse  : ACAGTAGGGAAATAAACTGTAAAGAAACT-AACAAGTGTTT-AACACTTTATTTTTACGT : 1719
Mouse  : -------AGAAGTCGAGTAAAAAGGAATT-AACAAGTGTTC-AAC--------------- : 1976
Rat    : -------AAAAGCCAACTGTAAGGAAATT-AACAACTGTTT-AACACATTCATTGTCTCT : 2012
                  AAat aAc   aA      T AaC     tTt AA a  t   t

                  *      2420         *      2440         *      2460
Human  : ------------------AGTCAAGTGAAA-GTATGAAAATGAGTTGAGGAAAGGCGAGT : 1665
Chimp  : ------------------AGTCAAGTGAAA-GTATGAAAATGAGTTGAGGAAAGGCGAGT : 1662
Rhesus : ------------------GGTCAAGTGAAAAGTATGAAAATTAGTTGAGGAAAGGTGAGT : 1642
Cow    : -------------------GTCAA---AACGGTATGAAATAGAATAGAGGGAAGGGGAGC : 1804
Dog    : -----------------AAGTCAAGTA-------TGGAACTGAGCCGA-----GGGAAGT : 2107
Cat    : TTTTCTTTTTTAAACGTATGTCAAGTAAAA-GTATGAAAGTGAATTGACGGAAGGGAAGC : 1752
Horse  : -----------------ATGTCAAGTAAAA-GTACGAAACTGAATGGAGAGGAGGGACGT : 1761
Mouse  : ---TCTCTCTGTCAGCTCTTTTTATTACGA--ATTCCAAGTAAAACTATGAACGGAAAGT : 2031
Rat    : CCCTCTTTCAGTGAGCTCTCTTTATTACGA--AATCCAAGTAAAAGTATGAAAGGAAAGT : 2070
                            gTcaA t   a   atg AA t A   gA g aaGG  aGt

                  *      2480         *      2500         *      2520
Human  : ACGTGGGTCAAAGCTGGGTCTGAGGAAAGGCTCACATTT--TGAGATCCCGACTCAATCC : 1723
Chimp  : ACGTGGGTCAAAGCTGGGTCTGAGGAAAGGCTCACATTT--TGAGATCCCGACTCAATCC : 1720
Rhesus : ACGTGGGTCAAAGCTGGGTCTGAGAAAAGGCTAACATTT--TGAGGTCCTGACTCAATCC : 1700
Cow    : ACGTGCGTCAGAGTTGCGTCTGAGGAAAGAC--ATTTCC--AGAGGTCCTGACACAATCC : 1860
Dog    : ACACGGGTCAGTGCTGGGTCTGAGGGCGGCCTGACATTTCCAGAGGTCCTGACGCAGTCC : 2167
Cat    : ACTTGGGTCAGTGCCGGGTCTGAGGGAGGACTAACATTTGCAGAGGTCCTGACACAATCC : 1812
Horse  : ATATGGGTCAGTGCTGGGTCTGAGGAAGGACTAACATCTACAGAGGTCCTGACACAATCC : 1821
Mouse  : GTGAGGG---GGACT--GTCTGAGAAAGGGATAACATTTCCTGAAGTTCTGGCACACCCT : 2086
Rat    : GTGCGGGACAGCATT--GTCTGAGAAAGGGCTAACATTTCCTGAAGGTCTGGCACACTCC : 2128
         a   GgGtca  gctg GTCTGAG aa G ct AcaTtt   GAggtcCtGaC CA tCc

                  *      2540         *      2560         *      2580
Human  : A-TGTCCCTTAAAGGGCACAGGGT-GTCTCCACAGGGCCGCCCAAAATCTGGTGAGAGAG : 1781
Chimp  : A-TGTCCCTTAAAGGGCACAGGGT-GTCTCCACAGGGCCGCCCAAAATCTGGTGAGAGAG : 1778
Rhesus : A-TGTCCCTTAAAGGGCACAGGGT-GTCTCCACAGGGCCACCCAAAATCTGGTGAGAGAG : 1758
Cow    : ACTGCCCCTCCTAGCACACGGGGC-G-------------GCTCAAAATCTCCTGGGGAGG : 1906
Dog    : GCTGC-CCTCAGAGGGCACAGGAT-GTAGCCACAGGACAGCCCAAAAGCTCTTGCGGAGG : 2225
Cat    : GCTGTGTCTCACAGGACACAGGAT-GTATCCATAGGACAGACCAAAATCTCTTGAGGAAG : 1871
Horse  : ATTGTCCGTCAGAGGACACAGGAT-GTGTCAA--ATGCAGCCTAAAATCTCCTGGGGTGG : 1878
Mouse  : ATTGGCCCTTAAAAAGTACTCAAA-GTTCCCACAATACTTCCCCAAATCACTAGGTAGTA : 2145
Rat    : ATTGGCCCTTAAAAAGTACTCAAAAGTTCCCACAATGCTTCCCCAAATCGCTAGGTAGTA : 2188
         a TG cccT a Ag  cAC gg   Gt  cca a   c  cccaAAAtCt  tG g   g

                  *      2600         *      2620         *      2640
Human  : ---GGCGTAGACGC-CTCACCTTCTGCCTCTACGGGTCACAAAAGCCTGGGTCACCCTGG : 1837
Chimp  : ---GGCGTAGACTC-CTCACCCTCTGCCTCTACGGGTCACAAAAGCCTGGGTCACCCTGG : 1834
Rhesus : ---GGCGT-GACTC-CTCACCCTCTGCCTCTACAGGTCACAAAAGCCTGGGTCACCCTGG : 1813
Cow    : ---GGAGA----GC-ATTACTGTCACCTTCTAAGGGTCACACAAGCCCGG-TCACCACTG : 1957
Dog    : --GAGCGGCGTTACTCTCACCTGCTGCCTCTAAGGGTCACACAAGGCTGGGTCACCGTGG : 2283
Cat    : AGGGGCGGCGTTATTCTCACCTTCTGCCTCTAAGGGCCACACAAGCCTGGGTCACCGTGG : 1931
Horse  : --GGGCGGCGTTACTCTCATCCTCTACCTCTAAGGCTCACAAAAGCCTGGGTCACCGTGG : 1936
Mouse  : --GAGCGA--TTTATTTTGCATCCTAGCCATAAGAGTCCCAGAAGTCAGGGTCAATGTAG : 2201
Rat    : --TAGCGA--TTTATTTTGCACTCTGGCCATAAAAGTCCCAGAAGTCAGGGTCAGTGTAG : 2244
             GcG         T ac  tCt cctcTA gggtCaCA AAG C GGgTCAcc t G

                  *      2660         *      2680         *      2700
Human  : TTGCCACTGTTCCTAGTTCAAAGTCTTCTTCTGTCTAATCCTTCACCCCTATTCT----C : 1893
Chimp  : TTGCCACTGTTCCTAGTTCAAAGTCTTCTTCTGTCTAATCCTTCACCCCTATTCT----C : 1890
Rhesus : TTGCCACCGTTCCTAGTTCAAAGTCTTCTTCTGTCTAATCCTTCACCCCTATTCT----C : 1869
Cow    : TTGCCACTGTTACCAGTTTAAAGTCTTCGTCAATCTAATGTTTCACCTCTATTCT----T : 2013
Dog    : AGGCCACTGTTACTAGTTCAAAGTCGTCTTCAGTGTGATTCTTCACCTGTAGTCT----T : 2339
Cat    : TTGCCACTGTAACTAGTTCAAAGTCGTCTTCGATCTAATTCTTCACCTTTAGGCT----G : 1987
Horse  : TTGCCACTGTTACTAATTCAAAGTC---TCCAATCTAACATTTCACCTTTATTCT----T : 1989
Mouse  : TTATCACTGTTCCTAGATCAAAGTGTTCTCCAATGGAATTTATCACTTTTTTTTTACTTC : 2261
Rat    : TTATAACTGTTCCTACATCAAAGTGTTCTCCAATCGAAGTTATCACTTTAACTTC----C : 2300
         ttgccACtGTt CtAgtTcAAAGTc tct C  TctaAt  tTCACc  ta tct

                  *      2720         *      2740         *      2760
Human  : GCCTTCCACTCCACCTCCCGCTCAGTCAGACTG--CGCTACTTTGAACCGGACCAAACCA : 1951
Chimp  : GCCTTCCACTCCACCTCCCGCTCAGTCAGACTG--CGCTACTTTGA-------------- : 1934
Rhesus : GCCTTCCACTCCACCTCCCGCTCAGTCGGGCTCTCCGCTACTTTGA-------------- : 1915
Cow    : GTCTCCCACTCCACCTCCAGCTCCATTAGGCTCTGCCCCACTTAAAAACACAAAACAAAA : 2073
Dog    : GTCCCCTCCTCCACCCCCAGCTCAATGAGCCTCTGCCCCACTTAAACGAAC--------- : 2390
Cat    : GACCCGTACTCCACCTCCAGCTCAATTAGA-TGTGCCCCACTTAAAAAAAAAAAAAAAAA : 2046
Horse  : GTCCCCCACTCCACCTCCAGCTCAATCAGACTCTGCCCCACTTCAAAAAGAAAA------ : 2043
Mouse  : CCTCCCGCCGCGACTTGCAGTTCAAACAGGTCCCACGCCAGTGCCGTGAAAAA------- : 2314
Rat    : CCTCCCGCCGCGACTTCCAGTTCAATCAGGTCCCACGCCAGCGCAGTGAAAAA------- : 2353
         g c  c  CtCcACctcC GcTCa t aG  t   C C Actt  a

                  *      2780         *      2800         *      2820
Human  : AACCAAACCAAACCAAACCAAAC--CAGACC-AG---------ACACCCCCTCCCGC--- : 1996
Chimp  : ------ACCAAACCAAACCAAAC--CAGACC-AG---------ACACCCCCTCCCGC--- : 1973
Rhesus : -----------AACAAACCAAAC--CAAACC-AG---------ACATCCCCC----G--- : 1945
Cow    : ACCACCTCCGACCCAAGCCAGTC--CAGATC-TGGAGAAGTGAACACTCTTGTTCGCTAT : 2130
Dog    : ----CCTCCAACCCAAGGCAGCC--CAGACT-CGGATACGTGCATACACGTGA-CGCTAC : 2442
Cat    : AAAGCCTCCAACCCAAGCCAGCC--CAGACTTCGAATAAGTGAATACACTTGATCACTAC : 2104
Horse  : ----CTTCCAACCCAAGACAGCCAGCAGGATTCGGATACATGAATACACTTAATCGCTAC : 2099
Mouse  : ------TCTACCCAATGCCCCTC--CACTTCTGG-----TCGGGCCCCATTTTCGACAAC : 2361
Rat    : ------TGTACCCAAAGCCCCTC--CACTTCTGG-----TTGGGCCCCATTTTCAAAAAC : 2400
                c a  ccAa cCa  C  CA      G         a ac c       c

                  *      2840         *      2860         *      2880
Human  : -GGAATCCCAGAGAGGCCGAACTGGGATAACCGGATGCATTTGATTTCCCAC--GCCACT : 2053
Chimp  : -GGAATCCCAGAGAGGCCGAACTGGGATAACCGGATGCATTTGATTTCCCAC--GCCACT : 2030
Rhesus : -GGAATCCTAGCGAGGCCGAACTGGGATAACCGGATGCATTTGATTTCCCAC--GCCACT : 2002
Cow    : GGGAGGCCCTTTCACACGCCCTTGGCTTGACCCCCAGCCCTCACTAACGGAACGGGCGCT : 2190
Dog    : GCGAGCACCGTTCCCACGCCTCTG-CTTGACCTTCTACAGGCAAAGGCGCTCTGGCCCTC : 2501
Cat    : AGGAGGATCTTTCCCACGCCTCCA-CCTGACCATCTACAGGAATAGGCGCTCTGGCCCTC : 2163
Horse  : GGGAGGCACTTTCCCAGGCCTCCG-ACTT-CTCCCTACAGAAATTGGCGCTCTGGCCCTC : 2157
Mouse  : TGGAGGCCTTTTCCCATGTCCTCG-CTT--CCTCCTGTACGGAAGAGGCCTCTAGTCCCC : 2418
Rat    : TGGAGGCCTTTTCCCACGTCCTCG-CTT--CCTCCTGTACAAAAAAGGCCTCTAGTCCCC : 2457
          gGA  cc        c      g   T  Cc   t ca        c c c  G C

                  *      2900         *      2920         *      2940
Human  : GA-GTGCACCTCT--GCAGAAATGGGCGTTCTGGCCCTCGCGAGGCAGTGCGACCTGTCA : 2110
Chimp  : GA-GTGCACCTCT--GCAGAAATGGGCGTTCTGGCCCTCGCGAGGCAGTGCGACCTGTCA : 2087
Rhesus : GA-GTGCACCTCT--GCAGAGATGGGCGTTCCGGCCCTCGCGAGGCGGTGCGACCTGTCA : 2059
Cow    : CA-GGACCCTGCCAGGCCACGCCAAAAGGCGCGACCTCGGCCAG-CGGTGCGACCTGTCG : 2248
Dog    : GCCGGGCGACGCCAAGCGACGCCAAGCGCCGCCGCCTCGGCCAG-CGGCGCAACCTGTCA : 2560
Cat    : GCCAGGCGCCACGAAGCGGCGCGA----------CCTCAGCCCG-CGGTGCGACCTGTCA : 2212
Horse  : GCTGGCCAACGCGAAGGGGGGCAA----------CCTGGGCCAG-AACCGCGACCCGTCG : 2206
Mouse  : GC------------------------------GGCGGGC-ACAGGCAGCCAGACTTAGTA : 2447
Rat    : AC------------------------------GGCTGGCCATAGGCAGCACGACCTGTTA : 2487
         g     c    c   g                  Cc   gc aG c g gcgACctgtca

                  *      2960         *      2980         *      3000
Human  : CCGCCCTTCA-GCCTTCCCGCCCTCCACCAAGCCCGCGCACGCCCGGCCCGCGCGTC-TG : 2168
Chimp  : CCGCCCTTCA-GCCTTCCCGCCCTCCACCAAGCCCGCGCACGCCCGGCCCGCGCGTC-TG : 2145
Rhesus : CCGCCCTTCA-GCCTTCCCGCCCTCCGCCGAGCCCGCGCACGCCCGGCCAGCGCCTC-TG : 2117
Cow    : CCGCCCTTCCCGCCTTCCCGCCGGCCGCCGAGCCCGCGCACGCCAAGCCCGCGCGCC-TC : 2307
Dog    : CCGCCCTTCCCGCCTTCCCGCCCTCCACCGAGCCCGCGCACGCCGAGCCCGCGCGCC-TT : 2619
Cat    : CCGCCCTTCCCGCCTTCCCGCC-TCCAACGAGCCCGCGCACGCCTAGCCCGCGCGCA-GT : 2270
Horse  : CCGCCCTTCCCGCCTTCCCGCCCTCCGCCGAGCCCGCGCACGCCGAGCCCGCGCGCC-TT : 2265
Mouse  : CTGCCCTTTACGCTTTCCCGCCTTTCACCAAGTGCGCGCGC-CAAAGGGTGCGCCCTGAT : 2506
Rat    : CTACCCTTTGCGCTTTCCCGCCGTTCACCCAGCGCGCGCGC-CAAAGTATCTGCCCAGTT : 2546
         CcgCCCTTc  GCcTTCCCGCC tcC cC AGccCGCGCaCgCc  Gcc gcGC    t

                  *      3020         *
Human  : TCTTTCGACCCGGCACCCC--GGCCGGTTCCC- : 2198
Chimp  : TCTTTGGACCCGGCACCCC--GGCCGGTTCCC- : 2175
Rhesus : ACGTTCGACCCGAATCCCC--GGACGGTTCCC- : 2147
Cow    : ACGTTGCACTCGGCCCCCCCTGGCCCATCCAG- : 2339
Dog    : ACGTTGGGCCCGGCCCCC---AGCCGGTGCCTC : 2649
Cat    : ACGTGTGGCCCGGCCCCC---AGCCGGTTTCCA : 2300
Horse  : ACGTTGGGCCCGGCCCCCC--GGCCGGATCCC- : 2295
Mouse  : ACCCTGAGGCAGGTACCC--------------- : 2524
Rat    : ACCCCAAGGCAGGTACCC--------------- : 2564
         aC tt   cccGg  CCC    g c

```
